# Transcriptomes of six mutants in the Sen1 pathway reveal combinatorial control of transcription termination across the *Saccharomyces cerevisiae* genome

**DOI:** 10.1371/journal.pgen.1006863

**Published:** 2017-06-30

**Authors:** Xin Chen, Kunal Poorey, Melissa N. Carver, Ulrika Müller, Stefan Bekiranov, David T. Auble, David A. Brow

**Affiliations:** 1 Department of Biomolecular Chemistry, University of Wisconsin School of Medicine and Public Health, Madison, Wisconsin, United States of America; 2 Department of Biochemistry and Molecular Genetics, University of Virginia Health System, Charlottesville, Virginia, United States of America; Stanford University School of Medicine, UNITED STATES

## Abstract

Transcriptome studies on eukaryotic cells have revealed an unexpected abundance and diversity of noncoding RNAs synthesized by RNA polymerase II (Pol II), some of which influence the expression of protein-coding genes. Yet, much less is known about biogenesis of Pol II non-coding RNA than mRNAs. In the budding yeast *Saccharomyces cerevisiae*, initiation of non-coding transcripts by Pol II appears to be similar to that of mRNAs, but a distinct pathway is utilized for termination of most non-coding RNAs: the Sen1-dependent or “NNS” pathway. Here, we examine the effect on the *S*. *cerevisiae* transcriptome of conditional mutations in the genes encoding six different essential proteins that influence Sen1-dependent termination: Sen1, Nrd1, Nab3, Ssu72, Rpb11, and Hrp1. We observe surprisingly diverse effects on transcript abundance for the different proteins that cannot be explained simply by differing severity of the mutations. Rather, we infer from our results that termination of Pol II transcription of non-coding RNA genes is subject to complex combinatorial control that likely involves proteins beyond those studied here. Furthermore, we identify new targets and functions of Sen1-dependent termination, including a role in repression of meiotic genes in vegetative cells. In combination with other recent whole-genome studies on termination of non-coding RNAs, our results provide promising directions for further investigation.

## Author summary

The information stored in the DNA of a cell’s chromosomes is transmitted to the rest of the cell by transcribing the DNA into RNA copies or “transcripts”. The fidelity of this process, and thus the health of the cell, depends critically on the proper function of proteins that direct transcription. Since hundreds of genes, each specifying a unique RNA transcript, are arranged in tandem along each chromosome, the beginning and end of each gene must be marked in the DNA sequence. Although encoded in DNA, the signal for terminating an RNA transcript is usually recognized in the transcript itself. We examined the genome-wide functional targets of six proteins implicated in transcription termination by identifying transcripts whose structure or abundance is altered by a mutation that compromises the activity of each protein. For a small minority of transcripts, a mutation in any of the six proteins disrupts termination. Much more commonly, a transcript is affected by a mutation in only one or a few of the six proteins, revealing the varying extent to which the proteins cooperate with one another. We discovered affected transcripts that were not known to be controlled by any of the six proteins, including a cohort of genes required for meiosis.

## Introduction

The process of transcription termination has a profound influence on the population of RNAs present in a cell, and thus on a cell’s physiology. The mechanisms by which termination influences RNA levels are manifold. For example, termination-coupled RNA degradation culls nascent transcripts created by pervasive initiation. This culling process appears to be the main pathway for rendering many intrinsically bidirectional promoters functionally unidirectional [1]. Early termination of a messenger RNA, called transcription attenuation, provides a way to decrease protein output even when initiation of synthesis of that mRNA is constant or increasing [2–5]. Failure to terminate an antisense transcript can decrease the level of the corresponding sense transcript by transcriptional interference, or increase the level of the sense transcript by disabling termination-coupled gene silencing [6,7].

In eukaryotes, most transcripts are synthesized by RNA polymerase II (Pol II), and the termination pathway utilized by these transcripts largely determines their fate. Transcription of protein-coding genes is usually terminated by a pathway coupled to cleavage and polyadenylation of the nascent transcript, which stabilizes the mRNA and stimulates export to the cytoplasm and translation [8,9]. In the yeast *Saccharomyces cerevisiae*, termination of short, non-coding Pol II transcripts typically uses an orthogonal pathway that is mediated by the nuclear DNA/RNA helicase Sen1 and the RNA-binding proteins Nrd1 and Nab3 [10]. The Sen1-dependent termination pathway is coupled to addition of a short poly(A) tail by the TRAMP complex, which targets the RNA for exonucleolytic trimming or complete degradation by the exosome [11]. While the Sen1 pathway is known to act on short stable RNAs, such as small nucleolar RNAs (snoRNAs), as well as cryptic unstable transcripts (CUTs), its full range of targets and the trans-acting factors that recognize them have not been elaborated.

In this study, we performed whole transcriptome analyses of *S*. *cerevisiae* strains containing conditional mutations in genes encoding six different essential proteins implicated in Sen1-dependent termination: Sen1, Nrd1, Nab3, Ssu72, Rpb11 and Hrp1, and compared them to wild-type controls. We find that reduced Sen1 activity has the most pervasive effects, implying a central function for this helicase in the pathway. Decreased Nrd1 and Nab3 activity have the most similar effects, yet a substantial fraction of Sen1-dependent terminators are insensitive to mutations in either factor, suggesting that other RNA-binding proteins are capable of eliciting the pathway. A subset of Sen1 targets is sensitive to mutations in the Pol II subunit Rpb11, but not to mutations in the other factors, implying the presence of an alternative pathway for these transcripts. We also obtained evidence for an unexpected function of Hrp1 as a positive elongation factor on many protein-coding genes. Our results reveal an unanticipated complexity in the factor requirements of Sen1-dependent termination, reminiscent of the diversity of mechanisms for initiation of transcription by Pol II.

## Results and discussion

### Choice of mutant alleles for transcriptome analysis

**Fig 1:**
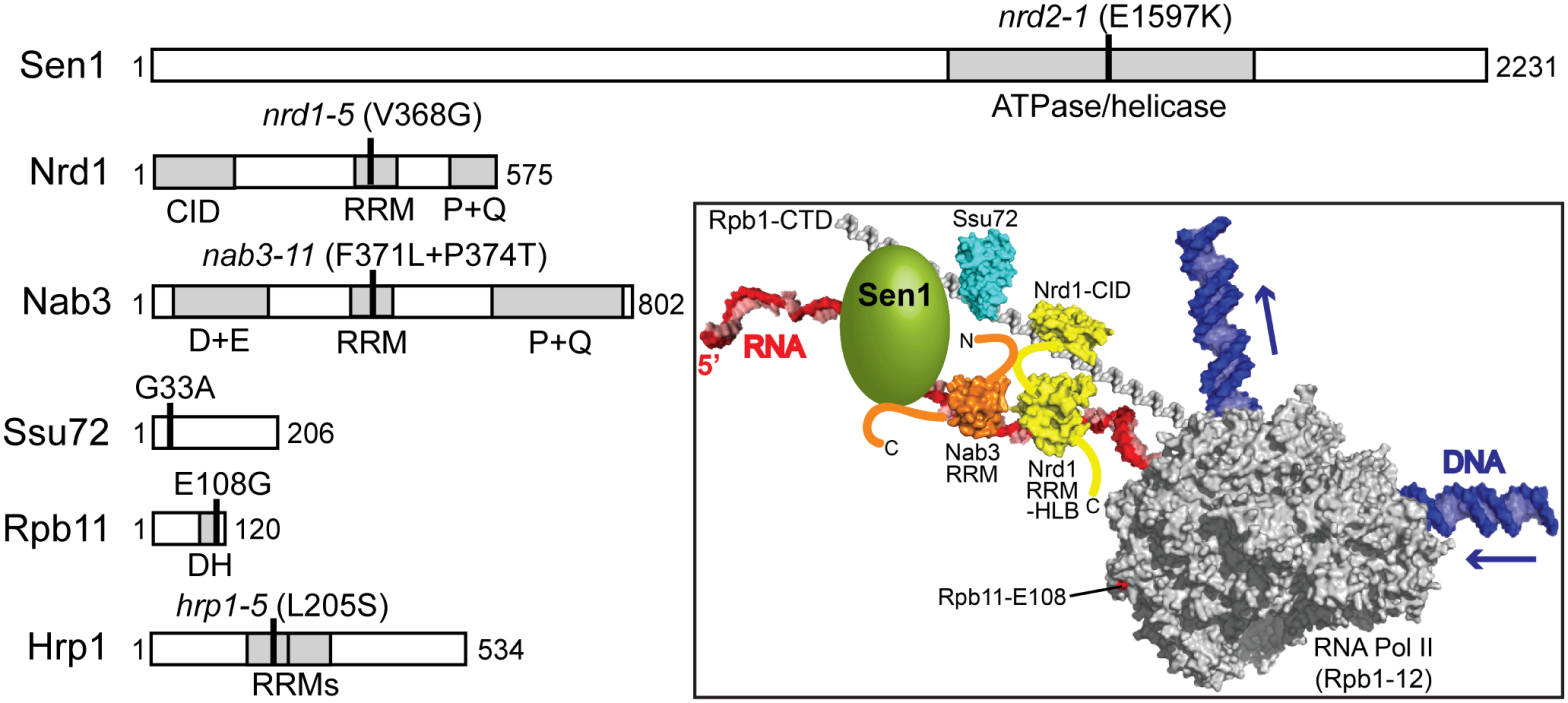
Mutations tested in this study for effects on transcript accumulation. Domain structures of tested proteins are shown to scale and sites of substitutions are indicated with heavy vertical lines. Numbers indicate amino acid residues. Domains (gray) are abbreviated as follows: CID, Rpb1 CTD-interacting domain; RRM, RNA recognition motif; P+Q, proline and glutamine-rich; D+E, aspartate and glutamate-rich; DH, dimerization (with Rpb3) helix. **Inset**: Model of the yeast Sen1-dependent termination complex. Yeast RNA Pol II (PDB ID: 1y1w) [28] is shown with the Rpb1 C-terminal repeats (PDB ID: 1sza) [82], DNA, and RNA modeled in. Also shown are the Nrd1 CID (PDB ID: 3clj) [23] and RRM/helix-loop bundle (PDB ID: 2m88) [16], the Nab3 RRM (PDB ID: 2xnq) [83], *Drosophila* Ssu72 (PDB ID: 3omw) [84], and Sen1, for which a structure of the helicase domain very recently became available [85]. Structures are not to scale and relative positions of proteins in the complex are arbitrary. Hrp1 is not shown.

We used temperature-sensitive alleles of six essential nuclear proteins to determine which transcripts are impacted by decreased activity of each. In two previous studies, the effects of nuclear depletion of either Nrd1 or Sen1 on the *S*. *cerevisiae* transcriptome were determined using the “anchor-away” method [12, 13]. A potential limitation of this approach is that associated proteins may also be depleted from the nucleus, or multiprotein complexes may be destabilized by the absence of one subunit, leading to uncertainty regarding the factor-specificity of the observed effects. Because we wished to compare the relative contributions of the six proteins, we chose instead to use well-characterized single or double amino acid substitutions to decrease their activities (Fig 1). For Sen1 and Nrd1, we used the *sen1-E1597K* (originally designated *nrd2-1*) and *nrd1-V368G* (originally designated *nrd1-5*) alleles obtained in the selection that identified the Sen1/Nrd1 termination pathway using an antisense terminator in the U6 RNA gene [14]. The Sen1-E1597K substitution appears to disrupt a salt bridge in the first RecA motif of the helicase domain [15] and alters the genome-wide distribution of Pol II even at the permissive temperature of 30°C [3]. The Nrd1-V368G substitution is in the RNA-binding face of the RNA recognition motif (RRM) [16]. Both mutations are lethal at 37°C (S1 Fig, see also S4 Fig) and result in 3’-extended transcripts from some snoRNA genes at restrictive temperature [17].

Two other alleles obtained in subsequent whole-genome selections for snoRNA gene terminator read-through were also tested: *ssu72-G33A* and *rpb11-E108G* [18, 19]. Ssu72 is a phosphatase that dephosphorylates serines 5 and 7 in the 26 seven-residue tandem repeats that comprise the C-terminal domain (CTD) of the largest subunit of Pol II, Rpb1 [20–22] (Fig 1). Nrd1 binds preferentially to serine 5-phosphorylated CTD repeats via its CTD-interacting domain [23], while Sen1 binds preferentially to serine 2-phosphorylated CTD repeats with an unidentified domain near the N-terminus of Sen1 [24] (Fig 1). The *ssu72-G33A* substitution alters a conserved glycine residue and is lethal at 37°C (S1 Fig). Other alleles of *SSU72* have also been observed to result in readthrough of some snoRNA (and mRNA) gene terminators [25–27]. Rpb11 is the second smallest subunit of Pol II, and Rpb11-E108 is a solvent-exposed residue on the upstream end of Pol II, opposite the DNA entry site [19, 28] (Fig 1). Adjacent substitutions in the C-terminus of the 120-residue Rpb11 also induce terminator readthrough, as does a lysine to glutamate substitution in residue 9 of Rpb3 [19], which is adjacent to Rpb11-E108 in elongating Pol II [28]. The mechanism by which mutations in this region of Pol II affect termination is unknown. The Rpb11-E108G substitution confers weak heat-sensitivity and strong cold-sensitivity. In this study, we tested its effects at 30°C.

Finally, we tested heat-sensitive substitutions in the RRMs of the RNA-binding proteins Nab3 and Hrp1 (S1 Fig, see also S4 Fig). Nab3-11 has two substitutions in its sole RRM, F371L and P374T [4, 29], while Hrp1-5 has a single substitution (L205S) in one of its two RRMs [30] (Fig 1). Nab3 forms a heterodimer with Nrd1 [29, 31, 32] and is thought to cooperate with Nrd1 to recognize many transcripts targeted by the Sen1 pathway, although this assumption has not been tested systematically. However, crosslinking of Nrd1 and Nab3 to the yeast transcriptome has been analyzed, and the two proteins co-localize to a broad spectrum of transcripts [33–35]. Hrp1 (also called Nab4) negatively autoregulates its expression by Sen1-dependent termination [3, 4], but it is unknown if it acts more generally as a Sen1 termination factor. Interestingly, a whole-transcriptome crosslinking study found that Hrp1 is associated with known targets of Sen1-dependent termination, including snoRNAs and CUTs, suggesting that Hrp1 may indeed act more generally in this pathway [36]. Yet, Hrp1 was first identified as a subunit of the mRNA cleavage and polyadenylation factor CF1 [30].

### Substitutions in different Sen1-related factors have surprisingly diverse effects on transcript accumulation

We used high-density tiled microarrays hybridized to double-stranded cDNA to measure the steady state level of transcripts across the genome in the six mutant strains relative to wild-type controls. With the goal of enhancing the termination defect, all strains were shifted from 30°C to 35°C for 45 minutes prior to RNA isolation, except the *rpb11-E108G* strain, which is only weakly heat-sensitive. (The wild-type control for *rpb11-E108G* also was not shifted.) Two biological replicates were done for each strain and the results were normalized and averaged (see Methods).

**Fig 2:**
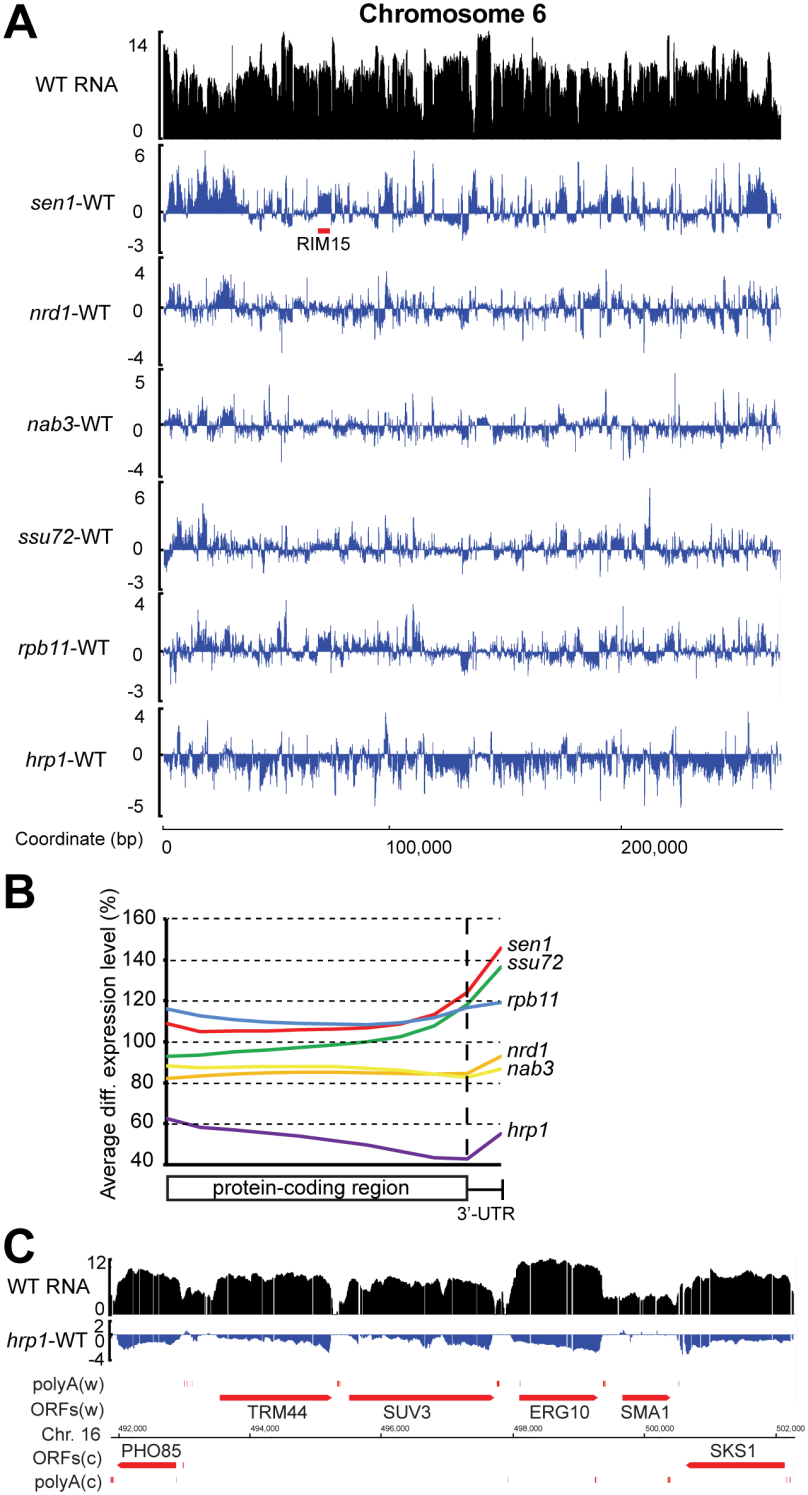
Overview of transcriptome changes in the six mutants. **A)** Low-resolution map of transcript changes across Chromosome 6. The vertical axis of each plot is log2 scale. The top plot (black) shows levels of RNA in the 46**a** wild-type (WT) strain. The lower six plots (blue) show the fold change in RNA level relative to WT in each of the mutants. The horizontal axis shows the position in base pairs from the left end of Chromosome 6. The position of the *RIM15* gene is shown by a red bar below the *sen1*-WT trace. **B)** Metagene plot of relative RNA level changes across the length of all annotated protein-coding genes in each of the six mutants. The values are relative to the matched wild-type strain. The horizontal axis represents the percent distance along each ORF and the first 100 bp of the 3’-UTR. **C)** “Sawtooth” pattern of changes in RNA level across six ORFs on Chromosome 16 in the *hrp1* mutant. Genes above the chromosome line (Watson or “w” strand) are transcribed to the right, and those below the line (Crick or “c” strand) are transcribed to the left.

To measure the genome-wide effects of the six mutations, we determined the fold change in RNA level over every probe in each mutant strain relative to wild-type and smoothed the log2-transformed values over a 101-base pair window. Fig 2A shows these data for Chromosome 6, which is short and thus allows a fairly high-resolution view, but is representative of the whole genome. The log2 RNA level for the wild-type 46**a** strain (WT) is shown in black and the log2 fold change in each mutant strain is shown in blue. It is evident that the net difference across the entire chromosome is positive for the *sen1-E1597K* strain and negative for the *hrp1-5* strain. The *sen1* mutant exhibits its most uniform increase in transcript accumulation in the subtelomeric regions, likely a reflection of the role of the wild-type protein in silencing these regions [3]. The ~ 5-kb continuous stretch of elevated transcript in the *sen1* mutant between 69 and 74 kb from the left telomere corresponds to the *RIM15* gene, which appears to be subject to Sen1-dependent attenuation (see below). The largely decreased transcript abundance in the *hrp1-5* strain across the chromosome (and the genome) is unexpected, and is discussed below. The other four mutants display a more balanced mix of increased and decreased transcripts. We deduce that we can detect (albeit, not precisely quantify) net changes in genome-wide Pol II transcript level without a spiked-in standard because the method we used for array normalization essentially normalizes to total RNA, most of which is made by Pol I and Pol III.

We used metagene analysis to quantify the effect of the six mutations on transcript accumulation across the length of all annotated protein-coding genes and 3’-untranslated regions (UTRs; approximated by the first 100 base pairs after the stop codon) relative to wild-type (Fig 2B). The *rpb11* mutant shows the greatest increase in transcript accumulation at the 5’ end of the open reading frame (ORF), followed by the *sen1* mutant. This order switches at the 3’ end of the ORF, where transcript accumulation peaks in the *sen1* mutant. The *ssu72* mutant has a pattern similar to *sen1*, except that it exhibits decreased transcript accumulation at the 5’ end of the ORF. This result is in agreement with a recent study by Zhang et al. [22], who observed decreased Pol II occupancy at the upstream end of protein-coding genes and increased Pol II at the downstream end upon depletion of functional Ssu72 [22], consistent with known functions of Ssu72 in both transcription initiation and termination [37].

Both the *nrd1* and *nab3* mutants exhibit a uniform decrease in transcript accumulation across the entire ORF with a modest uptick in the 3’-UTR. The *hrp1* mutant stands out as having sharply decreased transcript accumulation at the 5’ end of the ORF, with a steady further decrease along the length of the ORF, until the 3’-UTR. This phenomenon is clearly seen on a substantial number of individual genes, for example, on five of six adjacent genes on Chromosome 16 that display a “sawtooth” pattern in *hrp1* differential expression (Fig 2C). This observation implies that Hrp1 is a positive transcription elongation factor for mRNAs, consistent with RNA crosslinking studies that reveal occupancy of Hrp1 primarily in the promoter proximal region of mRNAs [36].

### Pairwise correlations suggest multiple pathways for Sen1-related factor function

Visual inspection of the transcriptome data revealed that, while readthrough of some Sen1-dependent terminators was induced by most or all of the mutations, other terminators were responsive to only one or a few mutations (see below). Furthermore, some changes in transcript accumulation were not obviously related to terminator read-through. To better define the functional overlap between the six factors, we calculated the degree to which changes in transcript level centered over each probe of the array (which are staggered at 5 base-pair intervals, on average) correlated for each pairwise combination of mutants (S2 Fig). We categorized the degree of similarity between pairs of mutants into 6 groups, based on the calculated Pearson's correlation coefficients and factor identity (Table 1).

**Table 1:** Correlation of effects on transcript accumulation for each pair of mutants.

Group	Mutant pair		Pearson's correlation coefficient
1	*nrd1*	*nab3*	0.66
	*sen1*	*nrd1*	0.64
	*ssu72*	*nrd1*	0.62
	*sen1*	*ssu72*	0.62
	*sen1*	*nab3*	0.61
2	*ssu72*	*nab3*	0.52
3	*nrd1*	*hrp1*	0.37
	*nab3*	*hrp1*	0.37
	*sen1*	*hrp1*	0.35
4	*sen1*	*rpb11*	0.34
5	*ssu72*	*hrp1*	0.24
	*ssu72*	*rpb11*	0.21
6	*nab3*	*rpb11*	0.17
	*nrd1*	*rpb11*	0.12
	*hrp1*	*rpb11*	0.08

Group 1 includes all pairwise combinations of Sen1, Nrd1 and Nab3, the three known components of the core termination complex. The Nrd1 and Nab3 mutants have the highest pairwise correlation coefficient of 0.66, consistent with the evidence that they function as a heterodimer. The Sen1 mutant highly correlates with the Nrd1 mutant and the Nab3 mutant, with the pairwise correlation coefficients 0.64 and 0.61, which agrees with the model that the heterodimer of Nrd1 and Nab3 recruits and/or activates Sen1 for termination [10]. The pairs of Sen1-Ssu72 and Nrd1-Ssu72 also fall into this group, and both have a pairwise correlation coefficient of 0.62. This correlation agrees with evidence for direct binding of the phosphorylated Rpb1 CTD by Nrd1 [23, 29, 38, 39] and Sen1 [24, 40].

Group 2 is the pair of Nab3 and Ssu72, with a correlation coefficient of 0.52. No direct interaction has been identified between Nab3 and the Pol II CTD, which may explain the lower correlation between Ssu72 and Nab3 than that between Ssu72 and Nrd1 or Sen1. The lower correlation suggests that some effects of decreased Nab3 activity are independent of CTD-mediated Sen1-dependent termination.

Group 3 includes the combinations between Hrp1 and all three components of the core Sen1 termination complex (correlation coefficients 0.35–0.37). This moderate correlation suggests that Hrp1 has a significant influence on Sen1-dependent termination, consistent with a recent crosslinking study [36].

Group 4 is the pair of Sen1 and Rpb11. The correlation coefficient between these two mutants (0.34) is higher than that between the Rpb11 mutant and the mutants of Nrd1 or Nab3 (0.12 or 0.17), suggesting that there is a function of Sen1 and Pol II that is independent of Nrd1 and Nab3. Thus, we propose this pairing is distinct from Group 3, despite the similar correlation.

Group 5 includes the pairs of Ssu72 with Hrp1 or Rpb11, with correlation coefficients of 0.24 and 0.21, respectively. Because Ssu72 and Hrp1 are both associated with the cleavage and polyadenylation machinery [30, 41], the correlation between them may reflect their involvement in mRNA 3' end processing. It is possible that this pathway involves functional interactions between Ssu72 and Rpb11.

Group 6 includes pairwise correlations between the Rpb11 mutant and the mutants in Nab3, Nrd1 or Hrp1. The correlation coefficients are low (0.17, 0.12 and 0.08), suggesting the Rpb11 mutation affects transcripts that are often not sensitive to the mutations of the other three, and vice versa.

We conclude from this analysis that different transcripts are acted upon by different subsets of the six proteins under study.

### Loops emanating from pairwise difference plots identify strongly affected transcripts

**Fig 3:**
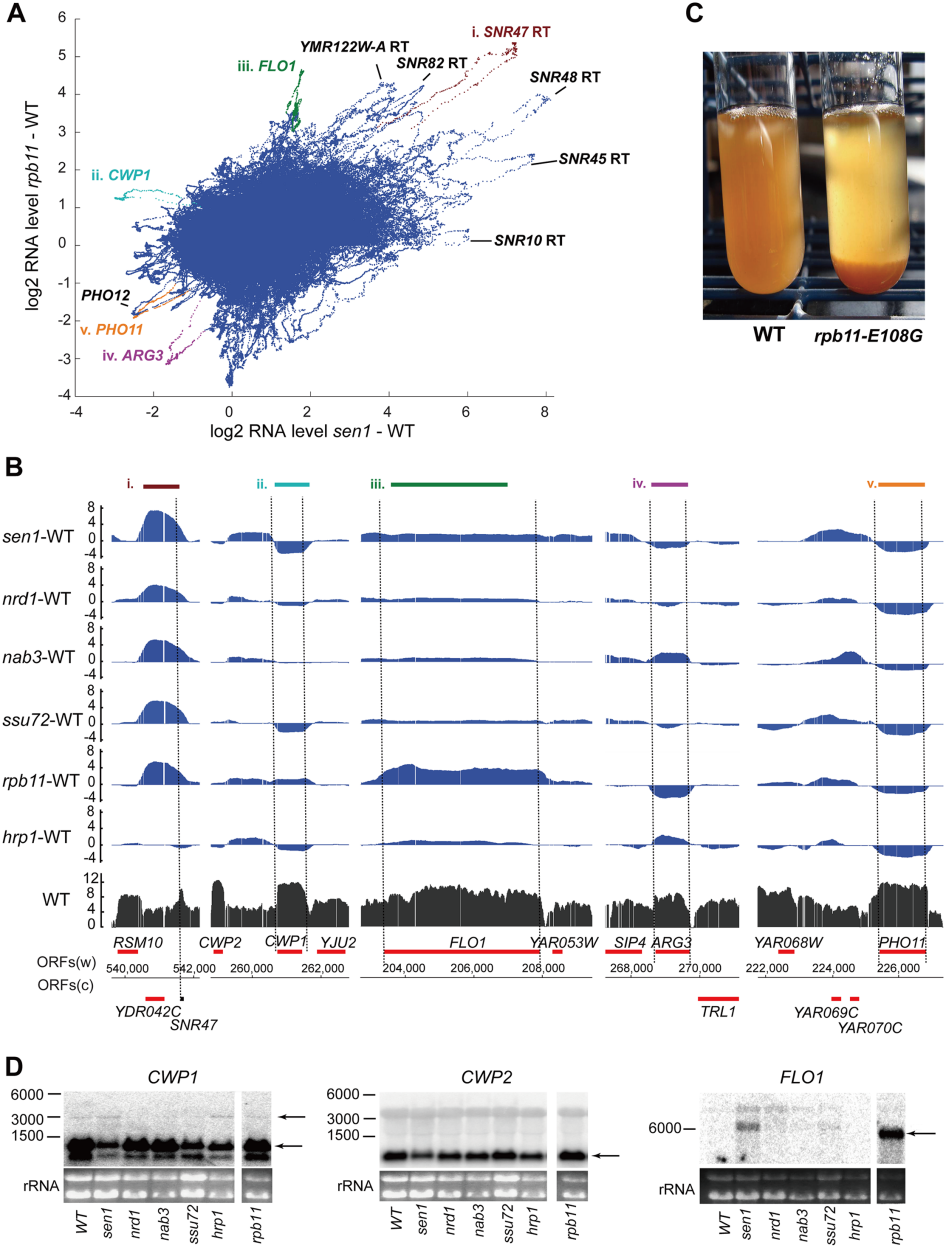
Discrete loops in pairwise difference scatter plots reveal strongly affected transcription units. **A)** Scatter plot of log2 fold change in RNA level relative to wild-type (WT) over every ~5 base interval in the *sen1* mutant (x axis) versus the *rpb11* mutant (y axis), using a 500 base pair smoothing window. Loops are labeled with the corresponding transcript. “RT” indicates a transcript arising from readthrough of the terminator of the indicated gene. Transcripts highlighted by a different color are shown in panel B. **B)** Changes in RNA level in all six mutants for the genes highlighted in panel A. The level of RNA in the wild-type 46**a** strain is shown in black at the bottom, aligned with the annotated genes. The upper six plots (blue) show the fold change in RNA level relative to WT in each of the mutants. See text for details. **C)** The *rpb11* mutant exhibits a strong flocculation phenotype, as indicated by rapidly sedimenting cells, relative to its wild-type parent, 46**a**. **D)** Validation of microarray results. Northern blots of total cellular RNA from haploid strains harboring wild-type (WT) and the indicated mutant alleles were probed for the listed transcripts. The positions of RNA markers of the indicated length in nucleotides are shown on the left, and the positions of the relevant transcripts are marked by arrows on the right. Uniformity of loading is indicated by the stained ribosomal RNA (rRNA) present in each lane, shown at the bottom.

Pairwise scatter plots made from data of such high resolution show an interesting feature when a long smoothing window (500 base pairs) is used, namely discrete loops that extend from the mass of data points (Fig 3A). These loops represent individual transcription units whose expression levels are most strongly altered by one or both mutations. In the case of the Sen1 vs. Rpb11 scatter plot, most of the loops corresponding to high differential expression in both mutants are snoRNA readthrough transcripts. However, the effect of the *rpb11* mutation on read-through of these terminators varies from high in the case of *SNR47* to low in the case of *SNR10* (Fig 3A and 3B,i). Also in this quadrant of the graph is a loop from readthrough of the putative mRNA for the 255 base pair YMR122W-A ORF, indicating that some short mRNAs may also be dependent on Sen1 and Rpb11 for efficient termination, as previously proposed [3].

Transcripts that are strongly decreased in the Sen1 mutant but not the Rpb11 mutant include the *CWP1* mRNA (Fig 3A and 3B,ii). We confirmed this result by Northern blot using a strand-specific *CWP1* probe (Fig 3D, left). In good agreement with the microarray data, the *CWP1* transcript is less abundant in the *sen1*, *ssu72*, and *hrp1* RNA samples. The *sen1* mutant displays increased transcript upstream of *CWP1*, which could be a readthrough transcript from *CWP2* that interferes with transcription of *CWP1* (Fig 3B,ii). However, a Northern blot probed for *CWP2* transcript shows no evidence of readthrough products (Fig 3D, middle), and the *ssu72* mutant displays decreased *CWP1* transcript in the absence of increased intergenic transcript, suggesting some other mechanism for decreased *CWP1* transcript. There is an ~ 3 kb transcript that is weakly detected by the *CWP1* probe and correlates reasonably well in relative abundance to the intergenic transcript (Fig 3D, left), which could thus arise from a transcription start site just downstream of *CWP2* and extend to the *CWP1* poly(A) site.

Genes whose transcripts increased much more in the *rpb11* mutant than the *sen1* mutant include *FLO1* (Fig 3A and 3B,iii), expression of which induces flocculation. The increased expression of the *FLO1* gene is indeed accompanied by flocculation (aggregation) of the *rpb11* mutant strain (Fig 3C). The strong increase in *FLO1* mRNA in the *rpb11* mutant and modest increase in the *sen1* mutant were confirmed by Northern blot (Fig 3D, right). The *FLO1* gene is located near the right telomere of Chromosome 1, which exhibits nearly global induction (or derepression) of transcription in the *rpb11* mutant. *FLO1* and some other subtelomeric genes are repressed by the Cyc8-Tup1 complex [42], so it is possible that *rpb11-E108G* mutation in Pol II diminishes the action of this complex. In contrast, the *rpb11* mutant decreased the expression of every gene in the arginine biosynthesis pathway, as well as the gene for the regulatory protein Arg80, while the *hrp1* and *nab3* mutants increased the expression of some of the *ARG* genes (Fig 3A and 3B,iv; S3 Fig). Genes encoding the two repressible acid phosphatase paralogs, *PHO11* and *PHO12*, which are both subtelomeric, exhibited decreased mRNA levels in response to all six mutations (Fig 3A and 3B,v).

Identification of the transcripts represented by the loops in scatter plots is a quick way to pinpoint major effects of the mutations under study. However, many other transcription units that are significantly affected are buried in the main body of the scatter plot. We used a custom MATLAB script to identify annotated transcription units with transcripts that are substantially altered by any of the six mutations. Several classes of such genes are discussed below, starting with the best-known targets of the Sen1 pathway, snoRNA and snRNA genes.

### sn/snoRNA genes exhibit differential sensitivity to mutations in Sen1-related factors

To quantify the effect of each mutation on the efficiency of termination of a given sn/snoRNA gene transcript, we calculated a “differential readthrough ratio” by dividing the average transcript level in a 50 base pair window 10 to 310 basepairs downstream of the mature sn/snoRNA-coding region by that of the sn/snoRNA-coding region itself, multiplying by 100%, and subtracting the corresponding value from the wild-type strain. This approach strikes a compromise between including unassociated downstream transcripts in the calculation and being fooled by endonucleolytic processing events that occur close to the mature snoRNA 3’ end (see below). In principle, a differential read-through ratio greater than 0% indicates a termination defect, although we considered values of less than 10% as indicating insignificant readthrough, as it was not detectable by Northern blotting (see below). We excluded from the analysis sn/snoRNA genes with closely adjacent or overlapping transcription units at their 3' ends that could contribute to the downstream signal, resulting in data for 47 genes (S1 Table). Nevertheless, in a minority of cases the differential read-through ratio is above 100%, possibly due to unannotated transcripts accumulating near the 3' ends of the sn/snoRNA genes in the mutants. The U6 snRNA gene (*SNR6*), which is transcribed by Pol III, acts as a negative control and did not exhibit readthrough. The only snoRNA gene transcribed by Pol III, *SNR52*, could not be analyzed because CUT567 is immediately downstream of it [43].

**Fig 4:**
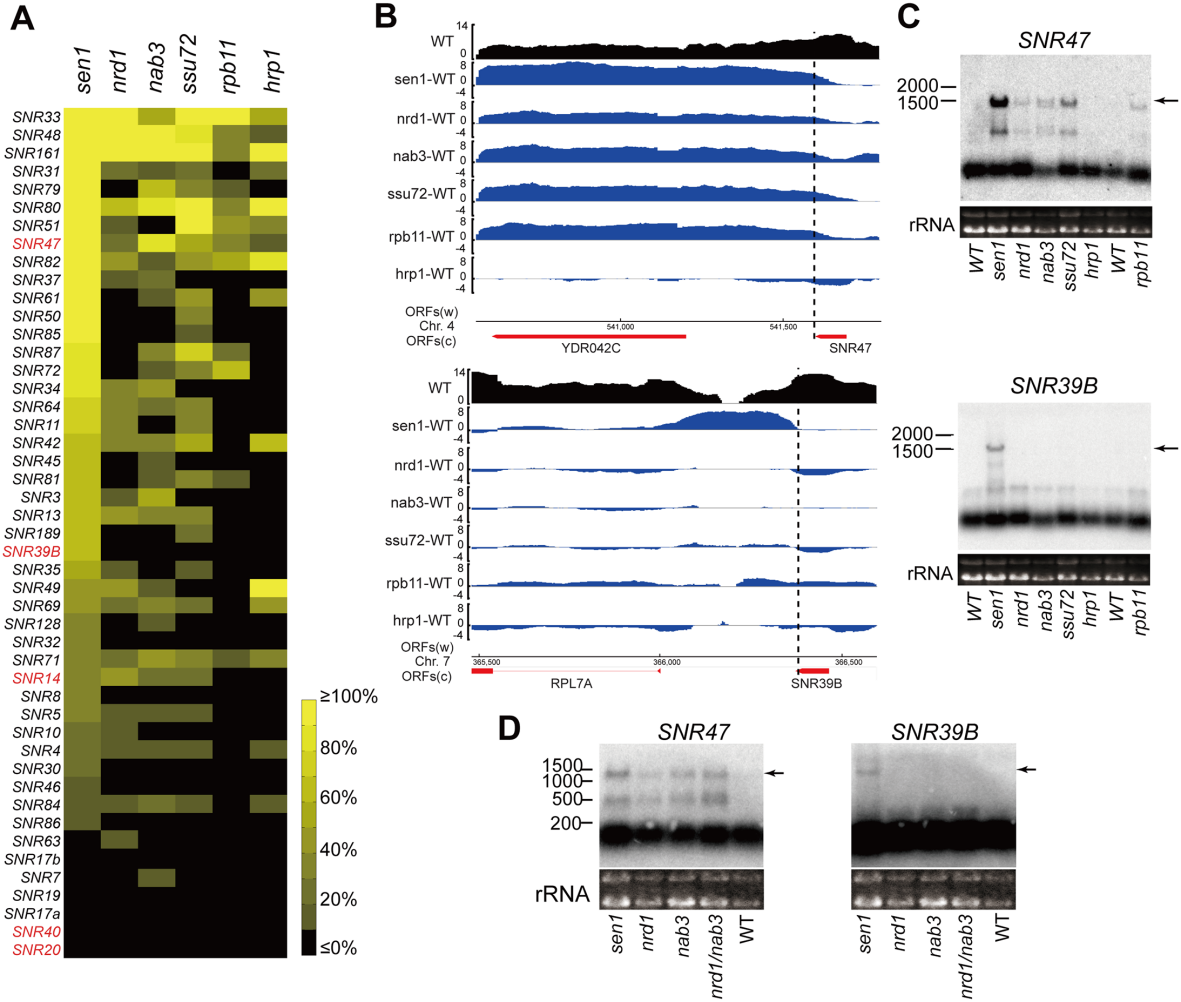
Effects of mutations on termination of transcripts from sn/snoRNA genes. **A)** A heat map shows the differential readthrough ratio (S1 Table) for the listed *SNR* genes in the six mutant strains ranked from high to low in the *sen1* mutant. (See scale at right.) The *sen1* mutant has the most pronounced effect on termination of sn/snoRNA transcripts. The genes marked in red are discussed in the text. **B)** Microarray data for *SNR47* and *SNR39B* is shown as log2 transformed values for the indicated genomic regions. Wild-type (WT) RNA level is black and the fold change in RNA level between each mutant and WT is blue. Dotted lines mark the mature 3’ end of snR47 (top) and snR39B (bottom). **C)** Validation of the array data by Northern blot, as in Fig 3, except here arrows indicate the longest readthrough transcripts. **D)** Nrd1 and Nab3 do not function redundantly on *SNR39B*. Northern blots of RNA from the indicated mutant strains were probed for *SNR47* and *SNR39B* transcripts. The longest readthrough transcripts are indicated with arrows.

Efficient termination of sn/snoRNA genes exhibited differential factor requirements (Fig 4A). For instance, the *SNR47* terminator exhibits readthrough in five of the six mutants, although to varying degrees (Figs 3B and 4B, top). Indeed, extended transcripts of *SNR47* were detected by Northern blot in all the mutants except *hrp1* (Fig 4C, top), for which the value of 11.4% readthrough is due to diminished mature RNA rather than increased downstream transcript. Northern blot analysis also agreed with the microarray-based finding that termination of *SNR39B* is only defective in the *sen1* mutant (Fig 4B and 4C, bottom). For both the *SNR47* and *SNR39B* Northern blots, intermediate bands were observed between the mature snoRNA and the extended product terminated at the downstream mRNA’s poly(A) site. These intermediate 3’ ends are likely due to intergenic terminators identified as poly(A) 3’ ends in a previous whole-genome study [44].

Of the six mutants, *sen1* showed the broadest influence, causing termination defects on 39 of the 47 analyzed *SNR* genes (Fig 4A and S1 Table). The only spliceosomal (sn) RNA gene that exhibited a termination defect in the *sen1* mutant is *SNR14*, which codes for U4 snRNA. Consistent with this finding, Sen1-dependent terminator elements have been identified downstream of *SNR14* previously, about 380 base pairs away from its transcription start site [17].

Most of the *SNR* genes that do not exhibit significant read-through in the *sen1* mutant produce primary transcripts and/or mature RNAs that are relatively long (>400 bp), such as *SNR20*, whose mature transcript has a length of 1175 nucleotides (Fig 4A, bottom and S1 Table). In agreement with this finding, Steinmetz *et al*. reported that *SNR20* did not exhibit terminator read-through in the Sen1 mutant based on Pol II ChIP [3]. This observation agrees with the notion that the Sen1 pathway preferentially terminates transcription of short genes (usually <450 bps) [3, 23, 45, 46]. However, *SNR40*, whose mature transcript is only 97 nucleotides long, shows no evidence of readthrough in any of the mutant strains. Chanfreau et al. [47] found that the primary transcript of snR40 is processed by the double-stranded RNA endonuclease Rnt1 and cotranscriptional Rnt1 cleavage can elicit termination [48, 49], which may explain the insensitivity of *SNR40* to the *sen1* mutant. The same argument applies to spliceosomal RNAs snR7/U5, snR19/U1, and snR20/U2, and to U3 RNA (snR17a/b), which are all processed by Rnt1 [50–53]. Yet, other snoRNAs that are processed by Rnt1, including snR39B and snR47 [47], do exhibit readthrough in the *sen1* mutant. Thus, the insensitivity of some sn/snoRNAs to *sen1-E1597K* is likely due to a combination of factors.

Transcription termination of sn/snoRNA genes is less often affected by the substitutions in the other five proteins. The *nrd1*, *nab3* and *ssu72* mutants cause termination defects on about two-thirds as many sn/snoRNA genes as *sen1*, on 25, 27, and 27 genes, respectively. Fewer sn/snoRNA genes show termination defects in the *rpb11* and *hrp1* mutants, 12 and 15 respectively. The identification of 15 snoRNA genes that are readthrough in the *hrp1* mutant suggests that it acts more generally as a Sen1-dependent termination factor, and is not restricted to its own mRNA.

A previous study that depleted Nrd1 from the nucleus using the anchor-away method reported that 80% of sn/snoRNA genes exhibit readthrough [12], which is higher than ~50% we observe that exhibit readthrough in the *nrd1-5* mutant, but very similar to the fraction of Sen1-dependent sn/snoRNA genes. This discrepancy could suggest that Nrd1-5 has residual function on some sn/snoRNA genes, and/or that nuclear depletion of Nrd1 destabilizes a complex containing the remaining Sen1-dependent termination factors, reducing their function. It is also possible that anchor-away of Nrd1 depletes associated factors such as Sen1 and Nab3 from the nucleus, so that all Sen1-dependent sn/snoRNA genes experience readthrough.

In summary, the activity of Sen1 is required for efficient termination of most short, Pol II-transcribed sn/snoRNA genes, while dysfunction of each of the other five proteins examined affects transcription termination of only a subset of Sen1’s target genes.

### Nrd1 and Nab3 are not functionally redundant for transcription termination of selected snoRNA genes

Our finding that substitutions in the RRMs of Nrd1 or Nab3 result in terminator readthrough on only a subset of Sen1-dependent snoRNA genes raised the possibility that Nrd1 and Nab3 have redundant functions on some snoRNA genes, such that mutation of only one does not result in terminator readthrough. To assess redundant effects of Nrd1 and Nab3 on transcription termination, we created a haploid strain that bears both *nrd1-5* and *nab3-11* alleles (see Methods). Previous efforts to create strains containing *nab3-11* and one of two partial deletion *nrd1* alleles resulted in cells that are either non-viable or very slow growing at 22°C, a temperature optimal for growth of a *nab3-11* single-mutant strain [32]. We found that the *nrd1-5*, *nab3-11* double mutant strain grows normally at 16°C and 23°C, but is lethal at 30°C and 37°C on a YEPD plate, similar to the *nab3-11* mutant alone (S4 Fig).

To look for redundant function, we compared transcripts of *SNR47* and *SNR39B* in the *nrd1-5* and *nab3-11* single mutant strains with those in the double mutant strain. In the case of *SNR47* (Fig 4D, left), terminator readthrough was detected in both single mutant strains as before, but was more severe in the double mutant, consistent with an additive effect of recognition by Nrd1 and Nab3 [54]. For *SNR39B* (Fig 4D, right), the extended transcript representing *SNR39B* read-through products was only detected in the *sen1* mutant, not in the *nrd1* and *nab3* single or double mutants. Thus, the absence of readthrough of the *SNR39B* terminator in the presence of *nrd1* or *nab3* substitutions is not due to functional redundancy of these two factors. Presumably, the *SNR39B* terminator requires an as yet unidentified specificity factor or Sen1 can recognize the terminator directly, perhaps via the RNA-binding activity of its helicase domain [55].

### Multiple-mutant analysis identifies a limited collection of attenuated mRNAs

The Sen1 pathway is known to regulate expression of a small number of proteins by transcription attenuation, that is, premature transcription termination in the 5’-UTR and/or protein-coding region. It is of interest to determine the factor-dependence of known targets of attenuation and to identify other yeast genes that are regulated in this fashion. For the latter, it is not sufficient to simply identify genes with increased transcript level in a mutant strain, since many such instances are due to readthrough of an adjacent Sen1-dependent terminator that is not regulated in a wild-type strain. Therefore, one must also look for features common to upstream regulatory transcripts.

**Fig 5:**
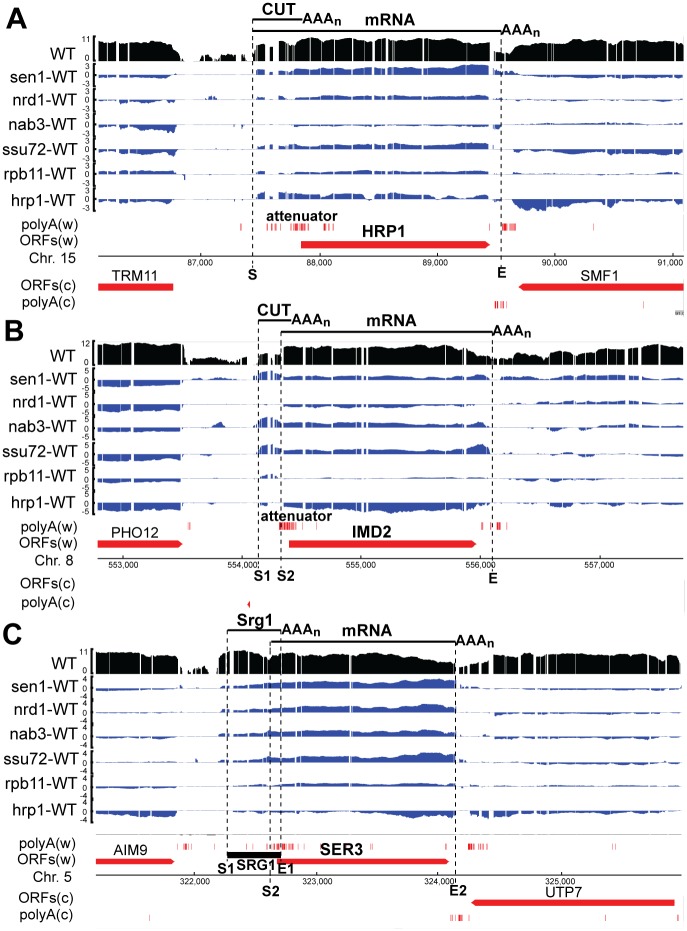
Three modes of regulation of mRNA synthesis by upstream transcripts. Each panel shows the log2 RNA level in the 46**a** strain (“WT”, in black) and the log2 fold change in transcript level (blue) in each of the six mutant strains. Annotated protein-coding regions (ORFs) and mapped poly(A) 3’ ends [44] on the Watson (w) and Crick (c) strands are shown in red. Approximate transcript 5’ (S) and 3’ (E) ends are indicated with dotted lines. **A)** The *HRP1* gene has a constitutive start site followed by a Sen1-dependent terminator (attenuator) that is responsive to the activity of Hrp1 and several other factors. **B)** The *IMD2* gene has an upstream start site (S1) that is used when the cellular GTP level is high, resulting in premature termination at the attenuator to create a CUT. Functional mRNA is produced from the downstream start site (S2) when the GTP level is low. **C)** The *SER3* gene is regulated by an independent upstream transcript, Srg1.

Three modes of regulation of yeast mRNA synthesis by upstream transcripts have been identified. The simplest involves a single TATA box and single cluster of transcription start sites (here called “ST-SS” for single TATA-single start), as observed in the *HRP1* and *NRD1* genes [2, 3, 17] (Fig 5A and S5A Fig). In ST-SS regulation, altered recognition of a Sen1-dependent terminator (attenuator) in a long 5’-UTR results in an altered proportion of stable mRNA and unstable CUT produced from the same start sites. A characteristic feature of ST-SS genes is the absence of ATG trinucleotides in the nontemplate strand between the transcription start site and the translation start site, despite their separation by hundreds of base pairs. This absence is necessary to prevent premature translation initiation as the ribosome scans through the long 5’-UTR. Also, appropriate terminator elements must be present in the long 5’-UTR to allow regulation, for example, Nrd1- and Hrp1-binding sites for autoregulation of *NRD1* and *HRP1*, respectively.

We find that full-length transcripts of *HRP1* accumulate in the *sen1*, *nrd1*, *ssu72*, and *hrp1* strains, increasing 3.5, 1.9, 2.3, and 1.9-fold, respectively (Fig 5A, S2 Table), consistent with autoregulation [3]. The *NRD1* gene (S5A Fig) exhibits strongly increased transcript abundance in the *sen1*, *nrd1*, and *nab3* strains (4.6, 3.1, and 3.0-fold, respectively; S2 Table), as determined previously [17]. Both genes display a low level of transcript accumulation in the *rpb11* mutant (1.6 to 1.7-fold). The mutation-induced transcripts generally terminate efficiently at the canonical poly(A) sites downstream of each gene, indicating that the mutations primarily affect Sen1-dependent termination. In a wild-type strain, premature transcription termination due to recognition of the attenuator results in a cluster of poly(A) 3’-ends at the upstream end of the ORF (Fig 5A and S5A Fig, “attenuator”), as detected previously by direct RNA sequencing [44]. These poly(A) 3’-ends presumably arise from Sen1-dependent termination-coupled polyadenylation by the TRAMP complex and are similar to the dense poly(A) site clusters observed downstream of snoRNA genes. The attenuated transcripts are not visible in the wild-type transcriptome data because they are rapidly degraded by the nuclear exosome and do not accumulate.

A more complex mechanism of regulation involves a single TATA box with dual sets of transcription start sites (ST-DS), as observed in the *IMD2* and *URA8* genes [3–5, 46, 56] (Fig 5B and S5B Fig). In this case, the transcription start site is regulated and transcripts that initiate at the more upstream sites (here labeled “S1”) encounter the attenuator and form CUTs, while transcripts that initiate at the more downstream sites (labeled “S2”) bypass the attenuator and become stable mRNA. For the *IMD2* and *URA8* genes, the concentration of the nucleotide synthesized by the gene product (GTP and UTP, respectively) determines transcription start site selection, resulting in feedback regulation [4, 5]. An increase in *IMD2* transcripts is observed in the *sen1*, *nab3* and *ssu72* strains (2.8, 3.0, and 3.7-fold, respectively; Fig 5B and S2 Table), consistent with a genetic screen for read-through of the *IMD2* attenuator that yielded mutations in *NAB3* and *SSU72* [27], and a similar selection that yielded a mutation in a consensus Nab3 binding site in the *IMD2* 5’-UTR [4]. *URA8* is similar to *IMD2*, accumulating full-length transcripts in the *sen1*, *nab3*, and *ssu72* mutant strains (2.7, 4.1, and 3.1-fold, respectively; S5B Fig and S2 Table). The *nrd1* mutant exhibits little or no increase in *URA8* mRNA and a decrease in *IMD2* mRNA, suggesting that Nrd1 is not involved in recognition of these attenuators, just as Nab3 appears not to be involved in recognition of the *HRP1* attenuator (Fig 5A). The fact that the *sen1* mutant exhibits strong transcript accumulation on all three genes (as well as *NRD1)* suggests a central function of Sen1 in attenuation. We did not see strong evidence for Sen1-dependent attenuation of the *ADE12* and *URA2* genes in our strains (S6A and S6B Fig), despite a previous report that these genes are regulated by a similar mechanism [5] and the presence of poly(A)-seq reads in their 5-UTRs. The reason for this apparent discrepancy is not clear.

A mechanism of regulation distinct from transcription attenuation, but that results in a very similar transcriptome profile, involves dual TATA boxes and dual transcription start sites (DT-DS), as illustrated by the *SER3* gene and its upstream regulatory RNA SRG1 [57, 58] (Fig 5C). In this case, an independent upstream transcript initiated from its own TATA box interferes with synthesis of the downstream mRNA, apparently by modifying the chromatin structure over its promoter [58, 59]. Read-through of *SRG1*, which occurs normally at a low level [60], increases strongly in the *sen1*, *nrd1*, *nab3*, and *ssu72* mutants, resulting in increased transcript spanning the *SER3* gene (5.1, 4.2, 3.7 and 4.6-fold, respectively; Fig 5C and S2 Table).

**Fig 6:**
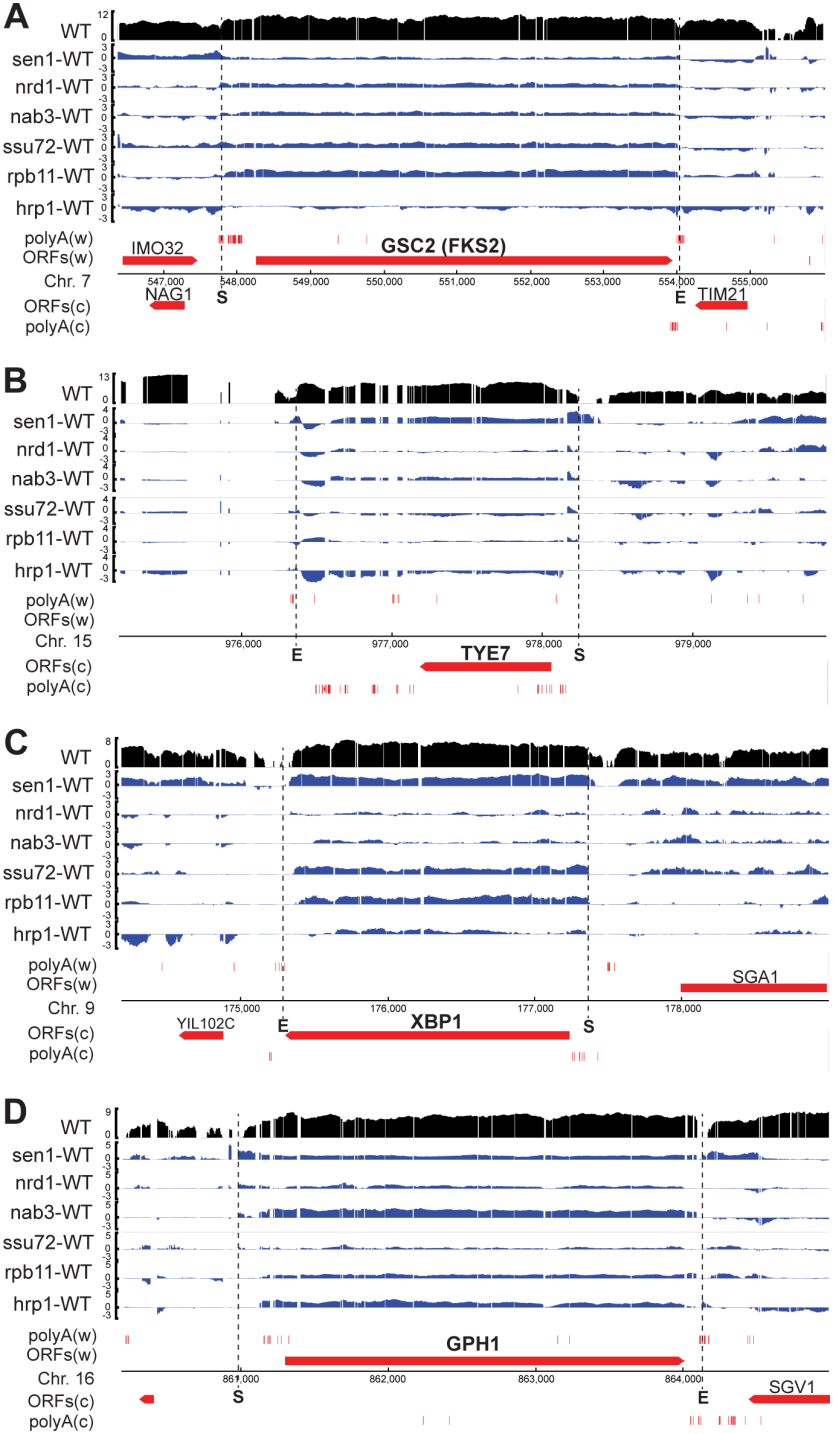
Factor-dependence of suspected and proposed attenuated genes. Changes in transcript levels over the indicated genes are displayed as described in the Fig 5 legend.

Using the properties of these examples of the three modes of regulation as search criteria, we manually inspected protein-coding genes that display increased transcripts in the *sen1* and other mutant strains (S2 Table) for additional possible examples. We were able to identify several genes that had previously been proposed by others to be regulated by Sen1-dependent termination (Fig 6). Kim and Levin [61] obtained evidence for Sen1-dependent attenuation of *GSC2* (also called *FKS2*). We observe modestly increased transcript of the *GSC2* gene in all of the mutants except *hrp1* (1.3 to 2.5-fold, Fig 6A and S4 Table). Surprisingly, we see the smallest increase in *GSC2* transcript in the *sen1* mutant and the largest increase in the *rpb11* mutant. Because the first upstream ATG trinucleotide is 504 base pairs upstream of the *GSC2* start codon, the attenuator read-through product is expected to be functional mRNA despite the very long 5’ UTR.

The *TYE7* transcript was identified as a binding target of Nrd1 and Nab3 by Darby et al. [62], and accumulates after nuclear depletion of Nrd1 [12]. We observe *TYE7* transcript accumulation of 2.6 and 1.7-fold in the *sen1* and *nab3* mutant strains, respectively, but only a 1.1-fold increase in the *nrd1* strain (Fig 6B, S2 Table), suggesting that Nrd1 nuclear depletion might act indirectly on this locus. We note that *TYE7* has several other features of ST-SS attenuated genes, including an upstream CUT that overlaps the ORF [63], a conserved TATA box-like sequence ~ 320 base pairs upstream of the start codon, the first upstream ATG ~ 250 base pairs upstream of the start codon, and a conserved consensus Nab3 binding site ~ 160 base pairs upstream of the start codon.

Darby et al. [62] also identified the *XBP1* transcript as a target of Nrd1 and Nab3, and showed that the *nrd1-102HA* mutant [29] accumulated 8-fold more *XBP1* transcript than an isogenic *NRD1HA* strain at a permissive temperature of 28°C. We observe 3.9, 2.4, and 2.6-fold accumulation of the *XBP1* transcript in the *sen1*, *ssu72*, and *rpb11* strains, respectively, but only a 1.1-fold excess in the *nrd1* strain and 1.3-fold excess in the *nab3* strain (Fig 6C, S2 Table). The *nrd1-102HA* mutant has a V379G substitution in the RRM as well as a C-terminal HA tag. The HA tag may disrupt functions of Nrd1 other than RNA binding, which could explain the stronger effect on *XBP1* attenuator readthrough than our *nrd1-V368G* mutant.

We were also able to identify a candidate attenuated gene that has not, to our knowledge, been noted before, namely *GPH1* (Fig 6D). *GPH1* codes for a glycogen phosphorylase [64], and has a consensus TATA box ~ 300 bp and a major transcription start site ~ 200 base pairs upstream of the start codon [65]. The first ATG sequence upstream of the ORF is upstream of the transcription start site and a consensus Nab3 binding site is ~ 70 base pairs upstream of the start codon. Thus, the *GPH1* gene has a long 5’ UTR that contains potential attenuator elements as well as poly(A)-seq reads (Fig 6D). We find that the *GPH1* transcript is elevated in all the mutant strains (1.3 to 4.4-fold, S2 Table) with the highest increase in the *nab3* mutant strain, consistent with the presence of a Nab3-binding sequence in the 5’ UTR. Two other potentially attenuated genes are *RIM15* and *SUL2*, which encode a protein kinase and a sulfate permease, respectively (Fig 2, S6C and S6D Fig, S2 Table).

Notably, the *NAB3* transcript also has a conserved Nab3 binding site ~ 70 base pairs upstream of the start codon, and exhibits 2.5-fold increased transcript abundance in the *nab3* mutant (S7 Fig and S4 Table). However, given the absence of similarly increased *NAB3* transcript accumulation in the *sen1* mutant, the increase may be due to a post-transcriptional effect of Nab3 binding on the stability of its mRNA. In this regard, it is interesting that an apparently Nrd1- and Sen1-independent function of Nab3 in recruiting the nuclear exosome subunit Rrp6 to RNAs was recently reported [66].

In summary, our transcriptome data are useful both for revealing the factor dependence of known mRNA targets of Sen1-dependent regulation and for identifying new targets of regulation. However, it is necessary to evaluate several different characteristics of a protein-coding gene to distinguish likely targets of regulation from genes that are subject to readthrough from nearby Sen1-dependent terminators in an unregulated fashion.

### Sen1 activity contributes to repression of meiotic gene expression in vegetative cells

**Fig 7:**
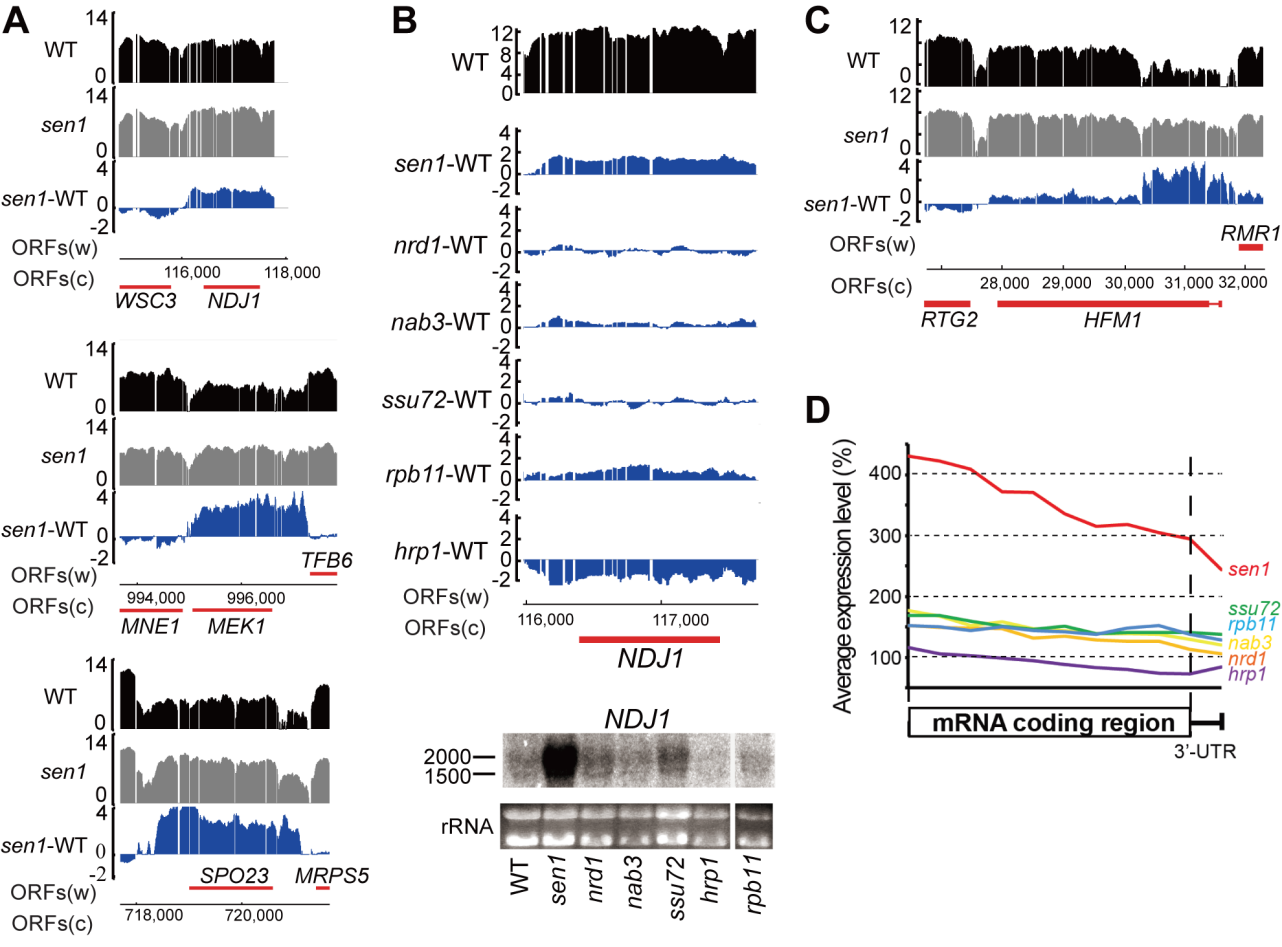
The *sen1* mutation induces accumulation of meiotic gene transcripts in vegetative cells. **A)** Accumulation of transcripts from the meiotic genes *NDJ1*, *MEK1*, and *SPO23* in a *sen1* mutant. The log2-transformed RNA level in wild-type (WT) and *sen1* mutant are shown in black and gray, respectively, and the fold change is shown in blue. **B)** Only the *sen1* mutant elicits strong accumulation of the *NDJ1* gene transcript, based on microarray data (top) and Northern blot (bottom). **C)** The *sen1* mutant activates the meiotic transcription start site for genes that experience internal initiation in vegetative cells. Microarray data for *HFM1* is shown. See S7 Fig for additional examples. **D)** Metagene plot of the average change in RNA level for the 69 genes listed in S3 Table in each of the six mutant strains. The first 100 nucleotides after the stop codon is used as a surrogate for the 3’-UTR. Only the *sen1* mutant exhibits a strong increase in RNA level, with a bias for the upstream end of the ORF.

Sen1 is known to repress Pol II transcription by at least two mechanisms: early termination (attenuation), as described above, and Pol II exclusion (silencing) coupled to Sen1-dependent termination of anti-sense transcripts. Targets of Sen1-dependent Pol II silencing include ribosomal RNAs, U6 RNA, and subtelomeric transcripts [3]. We searched for additional examples of protein-coding genes subject to Sen1-dependent silencing. There are 748 mRNA genes showing at least a two-fold net increase in the *sen1-E1597K* mutant (S2 Table), including those subject to attenuation or read-through of adjacent snoRNA genes and other non-coding transcripts. Gene ontology enrichment for all 748 mRNAs was performed using SGD YeastMine [67]. The “meiotic cell cycle process” GO term is the most enriched for the up-regulated mRNAs with 75 matches (p value = 3.8e-14), including *NDJ1*, *MEK1*, and *SPO23* (Fig 7A). In agreement with this finding, Sugiyama *et al*. reported that deletion of *S*. *pombe sen1*^*+*^ caused elevated mRNA levels from some meiotic genes [68]. (Note, however, that *sen1*^*+*^ is not essential in *S*. *pombe* and has a paralog, *dbl8*^*+*^, that is equally similar to *S*. *cerevisiae* Sen1.) To confirm that the 75 identified genes are indeed up-regulated during meiosis, we cross-referenced our list with a recent study of the *S*. *cerevisiae* meiotic transcriptome [69] (http://sgv.genouest.org/cgi-bin/viewer.cgi). At least 69 of the 75 genes are increased both during sporulation and in vegetative cells containing the *sen1* mutation (S3 Table).

We performed strand-specific Northern blot analysis to validate one of the up-regulated genes identified from the transcriptome data. The *NDJ1* transcript is strongly increased during sporulation [69] and in the *sen1* mutant based on our transcriptome data (Fig 7B, top). Using a strand-specific oligo probe, we confirmed that the *sen1* mutation causes significantly increased accumulation of the *NDJ1* transcript (Fig 7B, bottom).

Intriguingly, some meiotic genes exhibit a more significant increase in transcript from their upstream half than their downstream half, including *HFM1*, *MSH4*, *MSH5*, *ZIP1* (but not *ZIP2*), and *SSP1* (Fig 7C, S8 Fig). Published strand-specific transcript analysis confirmed that these genes are transcribed from internal transcription start sites in vegetative cells [70, 71], while increased transcript levels of their entire coding regions are observed during sporulation [69]. Thus, it appears that the upstream, meiosis-specific transcription start sites are derepressed in vegetative cells containing the *sen1* mutation. The effect seems to be *sen1*-specific, since metagene analysis of all 69 genes reveals strongly increased RNA accumulation with an upstream bias only in the *sen1* mutant (Fig 7D). It is therefore unlikely that the increased transcript levels over meiotic genes in the *sen1* mutant is primarily due to readthrough of flanking NNS-terminated transcripts into the transcriptionally inactive regions.

### Conclusions and prospects

The Sen1-dependent Pol II transcription termination pathway was discovered two decades ago, but it is clear that we still have much more to learn about its targets and functions. Here we compared the effects of mutations in genes encoding several different factors in the Sen1 pathway on the entire yeast transcriptome. The results obtained reveal an unexpected complexity in the factor requirements for Sen1-dependent termination on a broad range of targets.

The original genetic selection identified one specificity factor for the Sen1 pathway, a novel RNA binding protein that was named Nrd1 [14, 72]. A second specificity factor, Nab3, was identified both as a high-copy suppressor of a mutant Nrd1 protein and as a Nrd1-interacting protein in two-hybrid selections [29]. Mutations in Sen1, Nrd1 and Nab3 were subsequently shown to cause readthrough of certain sn/snoRNA gene terminators and of the first identified yeast attenuator, in the *NRD1* gene [17]. However, it was clear even from these early studies that not all snoRNA genes are sensitive to mutations in the Nrd1 RRM. The current study confirms and extends those early results, showing that while most snoRNA gene terminators experience readthrough when *SEN1* is mutated, many are insensitive to mutations in *NRD1* and/or *NAB3*. We show in one case that this is not simply due to redundant function, thus it is likely that specificity factors other than Nrd1 and Nab3 are used for Sen1-dependent termination, or that Sen1 can recognize some targets directly. In support of this hypothesis, we show that mutations in RRM1 of Hrp1 result in readthrough of a subset of Sen1-dependent terminators, although not the one that we found to be Nrd1- and Nab3-independent (*SNR39B*). There may well be other RNA-binding proteins that direct Sen1-dependent termination. A genome-wide selection for mutations that cause readthrough of the *SNR39B* terminator could potentially identify one or more such proteins.

The cohort of transcripts acted on by Sen1-dependent termination factors also is not well defined. Our study confirms the importance of this pathway for termination of genes encoding short, non-coding RNAs, but also identifies protein-coding genes that were not previously known to be influenced by Sen1-dependent termination. These genes include new potential targets of attenuation, such as *GPH1*, *RIM15*, and *SUL2*, as well as potential targets of Sen1-dependent silencing, such as the many meiotic transcripts whose abundance increases in vegetative cells carrying the *sen1-E1597K* allele. Our approach does not allow us to conclude whether the effect of the *sen1* mutant on meiotic transcripts is direct or indirect. We did not observe increased transcripts from the genes encoding the inducers of meiosis Ime1 and Ime4, both of which are normally repressed by noncoding RNAs in haploid cells [73, 74]. However, we did observe strongly increased accumulation of transcripts from the meiotic *ZIP2* gene in the *sen1* mutant strain (S8 Fig). The *ZIP2* gene is repressed in haploid cells by an antisense noncoding RNA [73], and it is possible that reduced Sen1 function interferes with antisense repression of *ZIP2* and other meiotic genes. Given the complexity of regulation of the meiotic program [73, 75], Sen1 could influence it in many different ways.

In combination with other published whole-genome studies on the Sen1-dependent termination pathway [12, 13, 24, 33, 34, 36, 76–78], our study on the effects of decreased function of Sen1 and its collaborating factors will illuminate the function of the Sen1 pathway in yeast cells.

## Methods

### Yeast strains

The yeast strains used for transcriptome analysis were derived from strains 46a and 46α as described previously [4, 14, 18, 19], and share the following genotype: *cup1Δ ura3 his3 trp1 lys2 ade2 leu2*. The unique genotypes of the mutant strains are as follows: *sen1-E1597K* (also called *nrd2-1*): *MAT***a** *sen1-E1597K*; *nrd1-V368G* (also called *nrd1-5*): *MAT*α *nrd1-V368G*; *nab3-F371L/P374T*: *MAT*α *nab3*::*KANMX* [pRS313*-nab3-11(F371L/P374T)*]; *ssu72-G33A*: *MAT*α *ssu72-G33A*; *rpb11-E108G*: *MAT***a** *rpb11-E108G*; *hrp1-L205S*: *MAT*α *hrp1*::*HIS3* [pRS315-*hrp1-5(L205S)*].

The Nrd1/Nab3 double mutant strain XCY377 (*MAT*α *cup1Δ ura3 his3 trp1 lys2 ade2 leu2 nrd1-V368G nab3*::*KANMX* [pRS313*-nab3-11(F371L/P374T)*])was created by integrating *nab3*::*KANMX* amplified from YSC1021-669432 (Open Biosystems) into the *nrd1-V368G* (*nrd1-5*) genome transformed with pRS316-NAB3 [4], followed by standard plasmid shuffle techniques. Integration at the *NAB3* locus was confirmed by PCR of genomic DNA with flanking primers and Sanger sequencing.

### RNA preparation

To make total cellular RNA for microarrays, yeast strains were grown in exponential phase for at least 2 generations in YEPD at 30°C to an OD600 of 1.0, then shifted to 35°C for 45 minutes and pelleted by centrifugation, except the Rpb11 mutant, which was grown continuously at 30°C. Total cellular RNA was prepared as described in [79]. For Northern blot analysis of the *nrd1/nab3* double mutant (Fig 4D), cells were grown in YEPD at 23°C to OD_600_ = 0.8–1.0, quickly shifted to 37°C by mixing with one volume of 51°C YEPD, incubated for 1 hour and pelleted by centrifugation. Total cellular RNA was prepared as above except that acid phenol, pH 4.3 (Fisher Scientific) was used for hot phenol extraction: 65°C for 6 minutes, vortexing every 2 minutes for 30 seconds.

### Northern blotting

For Northern blots, 15–20 μg total cellular RNA per lane was run on a 0.8% or 1% agarose/0.8 M formaldehyde gel and blotted to Zeta-probe membrane (Bio-Rad) by capillary transfer. USB OptiKinase (Affymetrix) was used to label oligonucleotide probes (sequences available by request) with [γ-^32^P]ATP (PerkinElmer) at 37°C for 1 hour. The probe was then separated from remaining [γ-^32^P]ATP by a G-50 Sephadex spin column (GE Healthcare). Blots were pre-hybridized in Church buffer (0.2 mM Na_2_HPO_4_, 0.1 mM NaH_2_PO_4_, 1 mM EDTA, 7% SDS, 10 mg/mL BSA) at 60°C for 1 hour, then ~ 10^6^ cpm/mL oligonucleotide probe was added and incubation continued for over 12 hours. After hybridization, blots were washed twice for 5 minutes at 23°C in 2× saline-sodium citrate (SSC), 0.1% SDS, and twice for 15 minutes at 42°C in 0.1× SSC, 0.1% SDS, and visualized with a Typhoon FLA 9000 (GE Healthcare).

### Genome-tiling microarray and data analysis

Transcriptome analyses of mutant and 46**a** and 46α wild-type strains used two independent biological replicates for each strain. cDNA synthesis from total cellular RNA, fragmentation, labeling, and hybridization to GeneChip *S*. *cerevisiae* Tiling 1.0R Arrays (Affymetrix) were done as described previously [80]. Briefly, the Affymetrix GeneChip Whole Transcript Double-Stranded cDNA Synthesis Kit was used as recommended by the manufacturer for model organisms. First and second strand cDNA was synthesized from random-sequence primers using dNTPs doped with dUTP. The product dsDNA was fragmented to an approximate length of 25 to 100 basepairs by treatment with uracil DNA glycosylase and apurinic endonuclease. The DNA fragments were then indirectly end-labeled with phycoerythrin. Raw data were generated by the Microarray Core Facility at the University of Virginia.

The genomic map (BPMAP) file in Affymetrix Tiling Analysis Software (TAS) version 1.1 was used to analyze intensity data in the raw microarray CEL files and evaluate the signal for each interrogated genomic position. Because the *S*. *cerevisiae* Tiling 1.0R Array is based on the reference genome sequence UCSC sacCer1 (released Oct. 2003) and there have been changes in later versions, the UCSC sacCer3 sequence (released Apr. 2011) was used to update the BPMAP file so that every probe on the chip that has a unique perfect match to the sacCer3 genome was assigned to its correct chromosomal location. The BPMAP file was updated by the function “remap” of the Bioconductor software package “Starr”.

The normalized and smoothed RNA levels of each sample were calculated following the TAS user guide available on the Affymetrix website. The intensities on the arrays of the two biological replicates were quantile normalized, and then the data were linearly scaled to a target median intensity of 100. The *S*. *cerevisiae* Tiling 1.0R Array has paired perfect match (PM) and mismatch (MM) probes, and MM probes serve as background. TAS applied the Wilcoxon Signed-Rank test to the normalized log2(max(PMi-MMi,1)) value whose genomic coordinate i fell within a designated 101-bp smoothing window to calculate the log transformed probability (-10log10(p-value)) that the RNA was detected above the noise level. The associated Hodges-Lehmann estimator was used to compute the signal intensity. If the estimate s of signal location is over 1, it was transformed to log_2_(s) for the RNA level. Otherwise, 0 was assigned for the RNA level.

Similarly, the differential RNA level was estimated by quantile normalizing replicate arrays of each strain and scaling the data to a target median intensity of 100 with mutant RNA samples as treatment and the matched wild-type RNA samples as control. Because both PM and MM probes are used to normalize mutant to wild-type RNA, and because the PM + MM signal is dominated by non-specific hybridization that presumably comes primarily from Pol I and Pol III transcripts that are highly abundant in total cellular RNA, changes in net Pol II transcript level are not expected to be negated by this normalization. TAS generated estimates of fold change by performing the Wilcoxon Rank-Sum test and calculating the associated Hodges-Lehmann estimator within a designated smoothing window. The difference between the treatment estimate s (s_t_) and the control estimate s (s_c_), st—s_c_, of signal location is interpreted as the log2 fold change between the treatment and control group signals. A 101-bp smoothing window was used for all the differential RNA level analyses, except as indicated otherwise.

The output RNA level and differential RNA level data are in the readable format with intensity value for each interrogated genomic position. All the genome features used for data visualization and computation in this study are downloaded from the *Saccharomyces* Genome Database (SGD, http://www.yeastgenome.org). Because the *HIS3* gene in the *nab3-F371L/P374T* mutant and the *LEU2* gene in the *hrp1-L205S* mutant are carried in plasmids, to avoid the interference from plasmid amplification data points that fall into the *HIS3* and *LEU2* regions (Chr15: 771,766–722,818 and Chr3: 91,185–92,702, respectively) were removed from these analyses. This process generated RNA level values of 2,585,209 locations across the whole genome for every experimental group. Integrated Genome Browser (IGB) [81] was used for data visualization. MATLAB (MathWorks, Inc.) was used for large-scale data computation.

## Supporting information

S1 Fig Serial dilution growth analysis of mutant strains on YEPD medium. The leftmost column at each growth temperature contains a spot of an overnight culture of the indicated strain diluted in YEPD medium to an optical density at 600 nm (OD600) of 0.11 to 0.13, followed by two successive 8-fold dilutions. The number of days of growth at the indicated temperature is shown for each panel. The poor growth of the *nrd1-5* strain at all temperatures may be due to its initial OD600 of 3.3 prior to dilution; the wild-type strains 46**a** and 46alpha were at a similar OD600 of 2.0 and 2.6, however Darby *et al*. [62] reported that Nrd1 may mediate the cellular response to glucose deprivation, so it may be more sensitive to nutrient depletion. See S4 Fig for more typical growth of the *nrd1-5* strain. The *rpb11-E108G* strain did not appear cold-sensitive in this experiment, although it did display its characteristic flocculence (as shown in Fig 3C).
(TIF)

S2 Fig Scatter plots of log2 fold change in transcript level relative to wild-type for all 15 pairwise combinations of mutants. Each data point represents the fold change in transcript level in the two indicated mutants for a ~ 5 basepair segment of the 12 x 10^6^ basepair yeast genome. The equation of the linear regression line and the Pearson’s correlation coefficient (R) are indicated on each plot. A value of 1 means perfect correlation, 0 means uncorrelated, and -1 means perfect anti-correlation. A smoothing window of 101 base pairs was used for these plots, which were generated by MATLAB.
(TIF)

S3 Fig Genes encoding enzymes in the arginine biosynthetic pathway and the regulator Arg80 are repressed in the *rpb11-E108G* strain. The data are displayed as in Fig 5, except that only the genomic region spanning each transcript is shown. Each gene comes from a different region of the genome, and genes are not to scale. The arrowhead at right indicates the *rpb11* track.
(TIF)

S4 Fig A *nrd1-5*, *nab3-11* double mutant grows similarly to the *nab3-11* mutant alone. Ten-fold serial dilutions of wild-type (WT) and the indicated mutant strains were plated to YEPD medium and incubated at the listed temperatures to similar cell densities for wild-type. The *sen1* mutant is shown for comparison.
(TIF)

S5 Fig Regulation of *NRD1* and *URA8* mRNA synthesis by attenuation. Changes in transcript levels over the indicated genes are displayed as in Fig 5.
(TIF)

S6 Fig The *ADE12* and *URA2* genes do not show evidence of Sen1-dependent attenuation, while the *RIM15* and *SUL2* genes do. The microarray data are displayed as in Fig 5.
(TIF)

S7 Fig Increased *NAB3* mRNA in the *nab3* mutant. Changes in transcript levels over the indicated genes are displayed as in Fig 5.
(TIF)

S8 Fig Some meiotic genes that have internal transcription start sites in vegetative cells preferentially accumulate upstream transcripts in the *sen1* mutant. *ZIP2* is the only gene shown that does not display an upstream bias in transcript accumulation in the *sen1* mutant.
(TIF)

S1 Table Differential read-through ratio (%) of sn/snoRNA genes in the six mutants. Percent change in the transcript level downstream of the indicated sn/snoRNA genes in mutant strains relative to wild-type was calculated as described in the text. U RNAs transcribed from *SNR* genes are listed after a slash. Positive changes of greater than 10% are considered indicative of terminator read-through and are in bold font. Changes of greater than 100% are likely due to overlapping transcripts from other sources.
(DOCX)

S2 Table Annotated list of protein-coding genes whose transcript levels increase at least 2-fold in the *sen1* mutant strain. The standard name is listed if one is assigned; cyan highlighting indicates known or potential attenuated genes. Genes induced in meiosis are noted under “process”; other cellular processes are not noted. Adjacent non-coding RNA genes transcribed towards the listed protein-coding gene and potentially contributing to the increased signal in the *sen1* mutant are noted, although this listing is incomplete. snoRNA genes are highlighted in yellow.
(XLSX)

S3 Table Genes induced in meiosis that are more than two-fold up-regulated in vegetative cells in the *sen1* mutant. Genes indicated in bold font are shown in Fig 7 or S8 Fig.
(DOCX)

S4 Table Effects of the six tested mutations on average transcript levels over 5794 protein-coding genes. The log2-transformed (left) or untransformed (right) fold change in average transcript level over all annotated ORFs in each mutant strain relative to wild-type is shown, ranked by decreasing effect of the *sen1* mutant.
(XLSX)
