# Supplementary figures and images for: Transcriptomes of six mutants in the Sen1 pathway reveal combinatorial control of transcription termination across the Saccharomyces cerevisiae genome
Source: PLoS Genet. 2017 Jun 30;13(6):e1006863. doi: 10.1371/journal.pgen.1006863 (PMC5513554; doi:10.1371/journal.pgen.1006863)

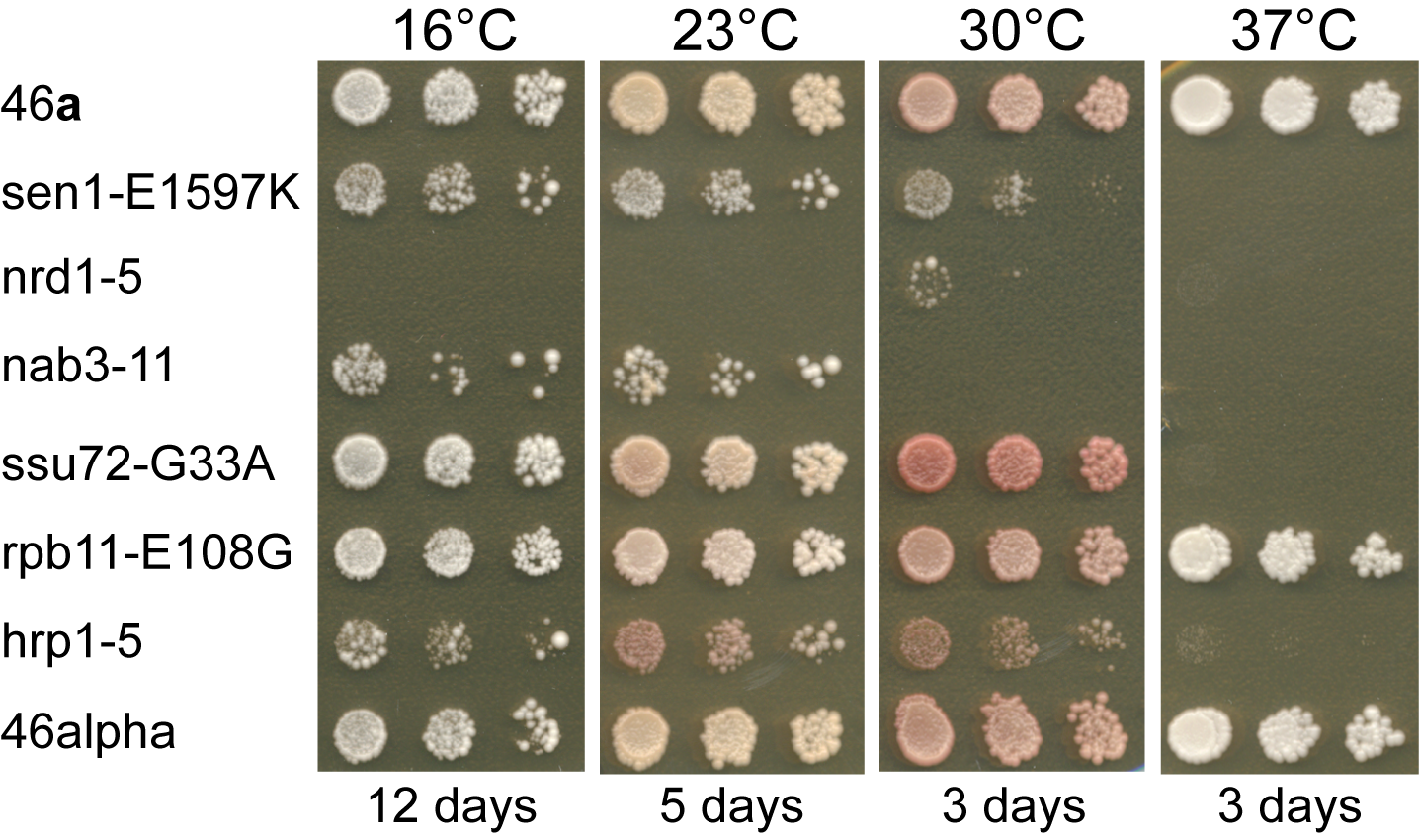

Supplement: S1 Fig — The leftmost column at each growth temperature contains a spot of an overnight culture of the indicated strain diluted in YEPD medium to an optical density at 600 nm (OD600) of 0.11 to 0.13, followed by two successive 8-fold dilutions. The number of days of growth at the indicated temperature is shown for each panel. The poor growth of the nrd1-5 strain at all temperatures may be due to its initial OD600 of 3.3 prior to dilution; the wild-type strains 46a and 46alpha were at a similar OD600 of 2.0 and 2.6, however Darby et al. [62] reported that Nrd1 may mediate the cellular response to glucose deprivation, so it may be more sensitive to nutrient depletion. See S4 Fig for more typical growth of the nrd1-5 strain. The rpb11-E108G strain did not appear cold-sensitive in this experiment, although it did display its characteristic flocculence (as shown in Fig 3C). (TIF) [file pgen.1006863.s001.tif]

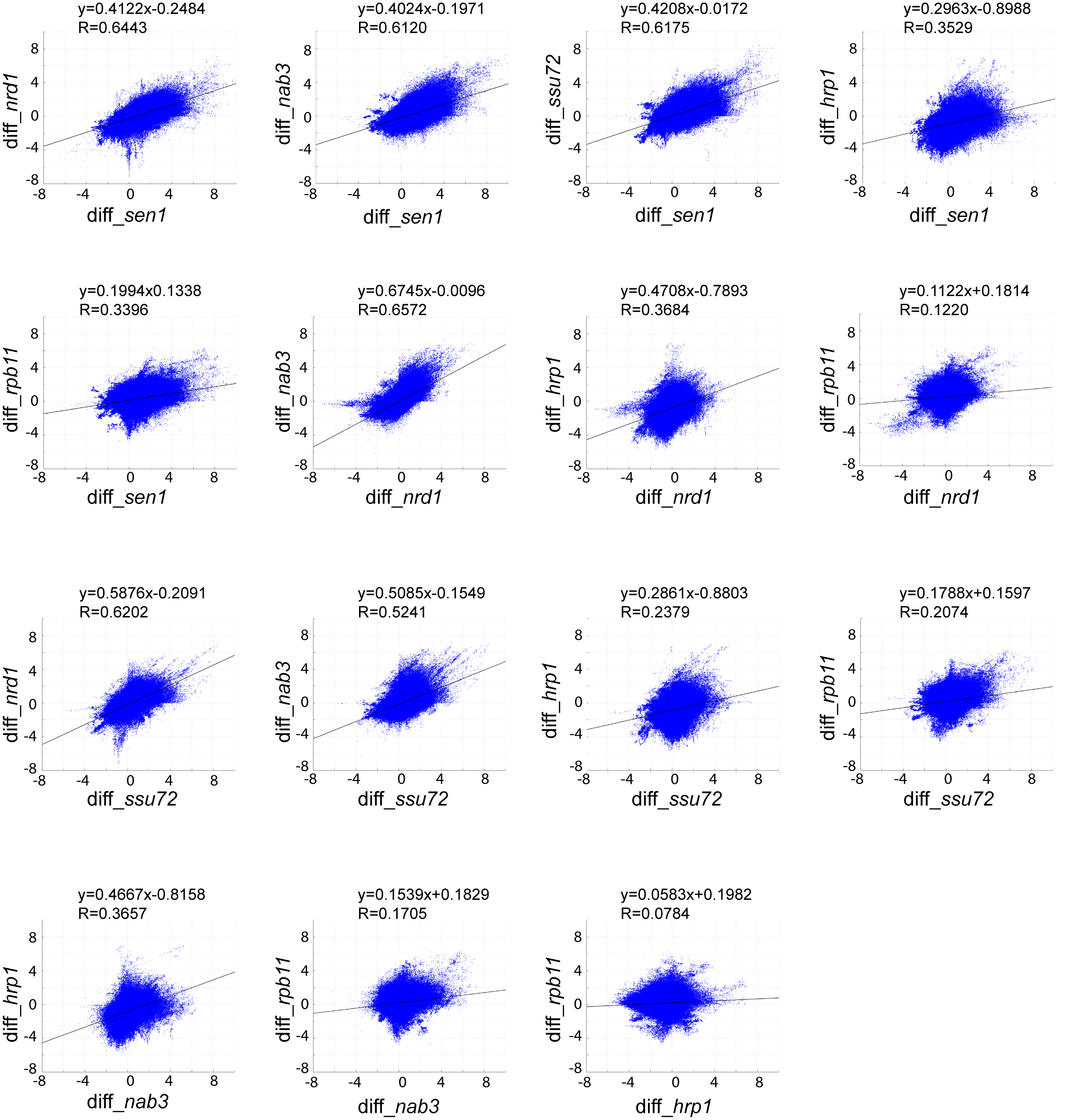

Supplement: S2 Fig — Each data point represents the fold change in transcript level in the two indicated mutants for a ~ 5 basepair segment of the 12 x 106 basepair yeast genome. The equation of the linear regression line and the Pearson’s correlation coefficient (R) are indicated on each plot. A value of 1 means perfect correlation, 0 means uncorrelated, and -1 means perfect anti-correlation. A smoothing window of 101 base pairs was used for these plots, which were generated by MATLAB. (TIF) [file pgen.1006863.s002.tif]

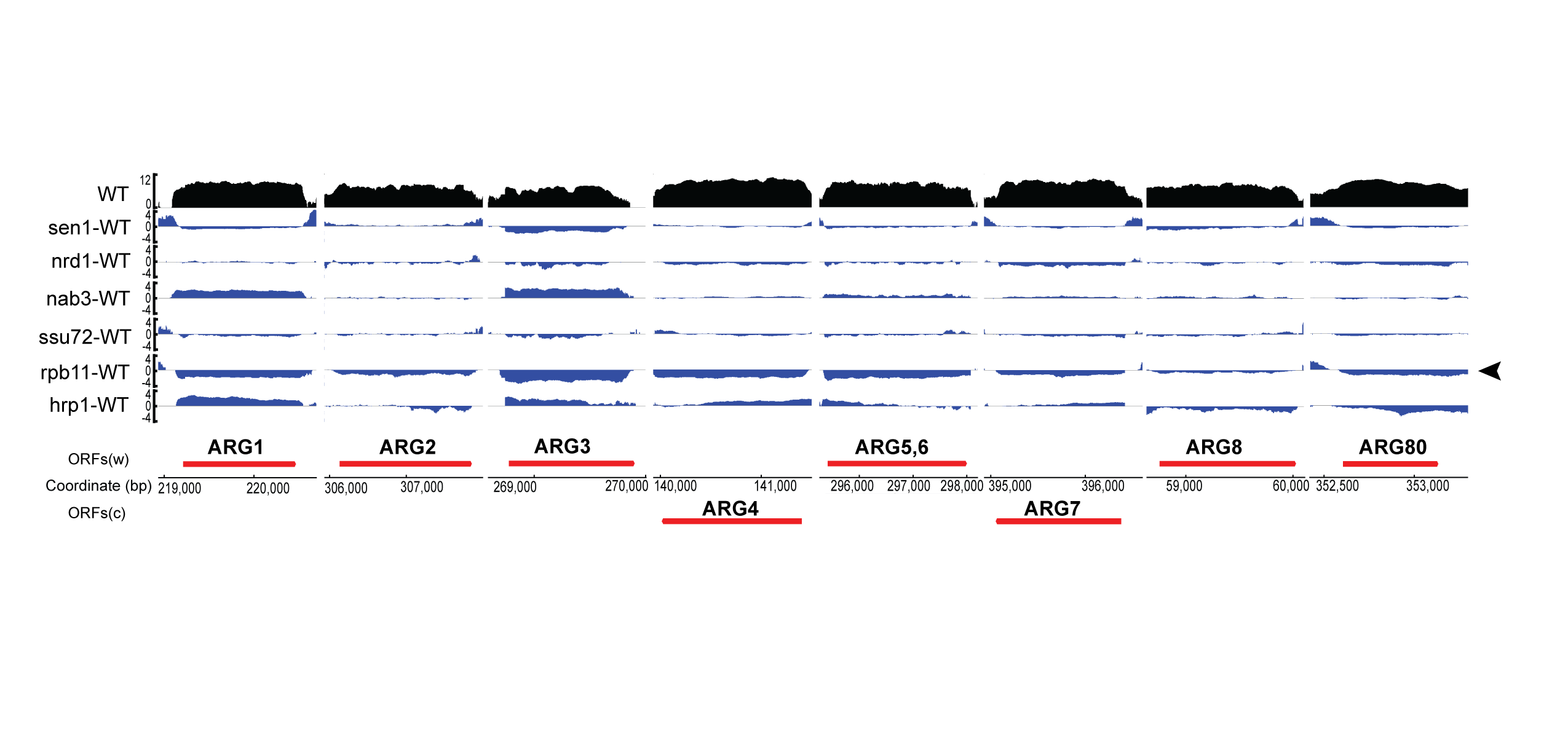

Supplement: S3 Fig — The data are displayed as in Fig 5, except that only the genomic region spanning each transcript is shown. Each gene comes from a different region of the genome, and genes are not to scale. The arrowhead at right indicates the rpb11 track. (TIF) [file pgen.1006863.s003.tif]

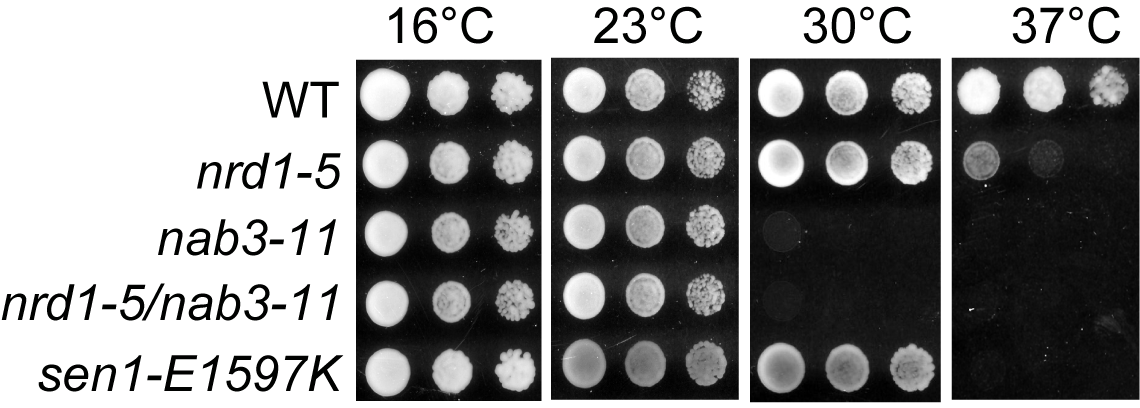

Supplement: S4 Fig — Ten-fold serial dilutions of wild-type (WT) and the indicated mutant strains were plated to YEPD medium and incubated at the listed temperatures to similar cell densities for wild-type. The sen1 mutant is shown for comparison. (TIF) [file pgen.1006863.s004.tif]

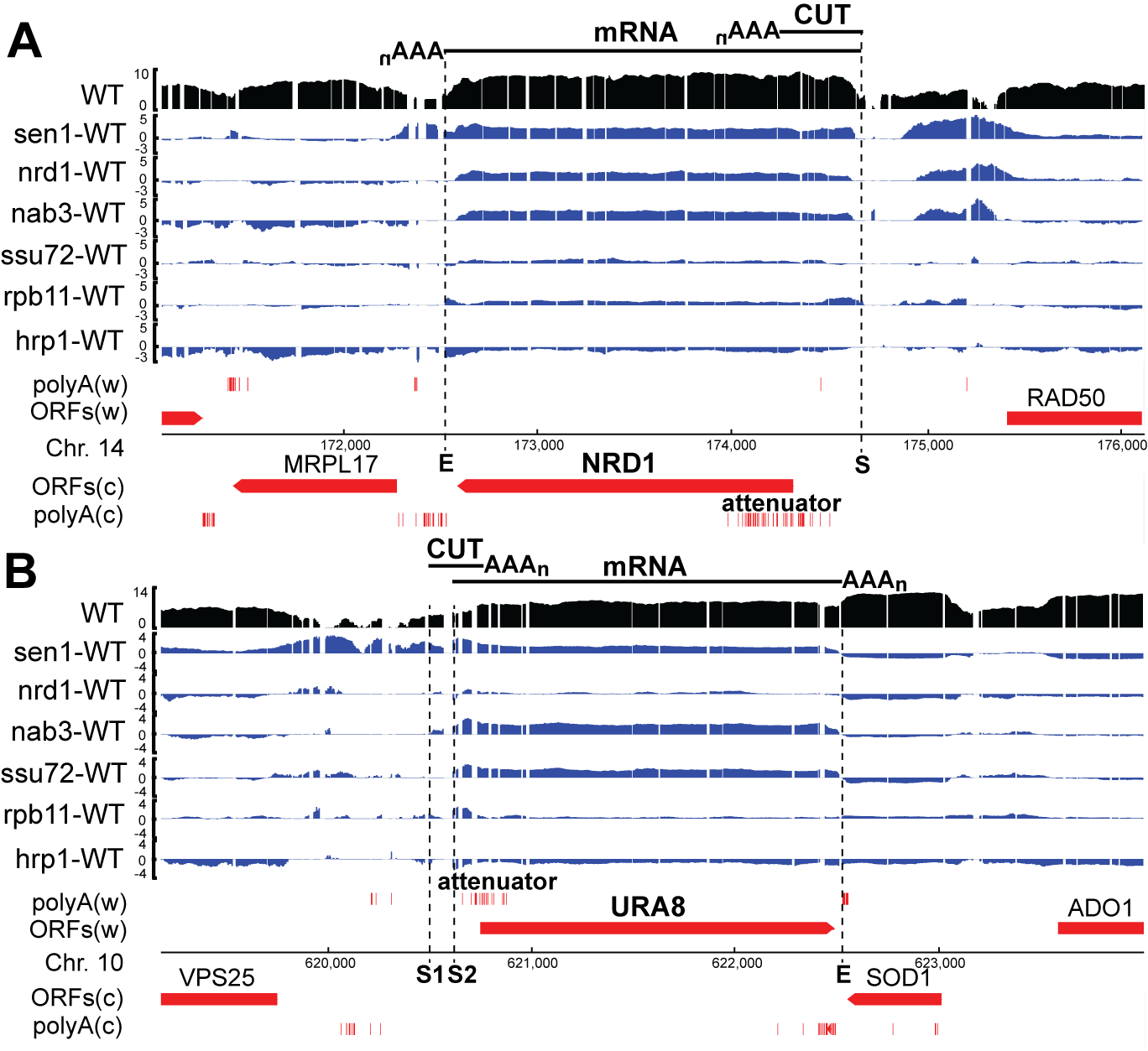

Supplement: S5 Fig — Changes in transcript levels over the indicated genes are displayed as in Fig 5. (TIF) [file pgen.1006863.s005.tif]

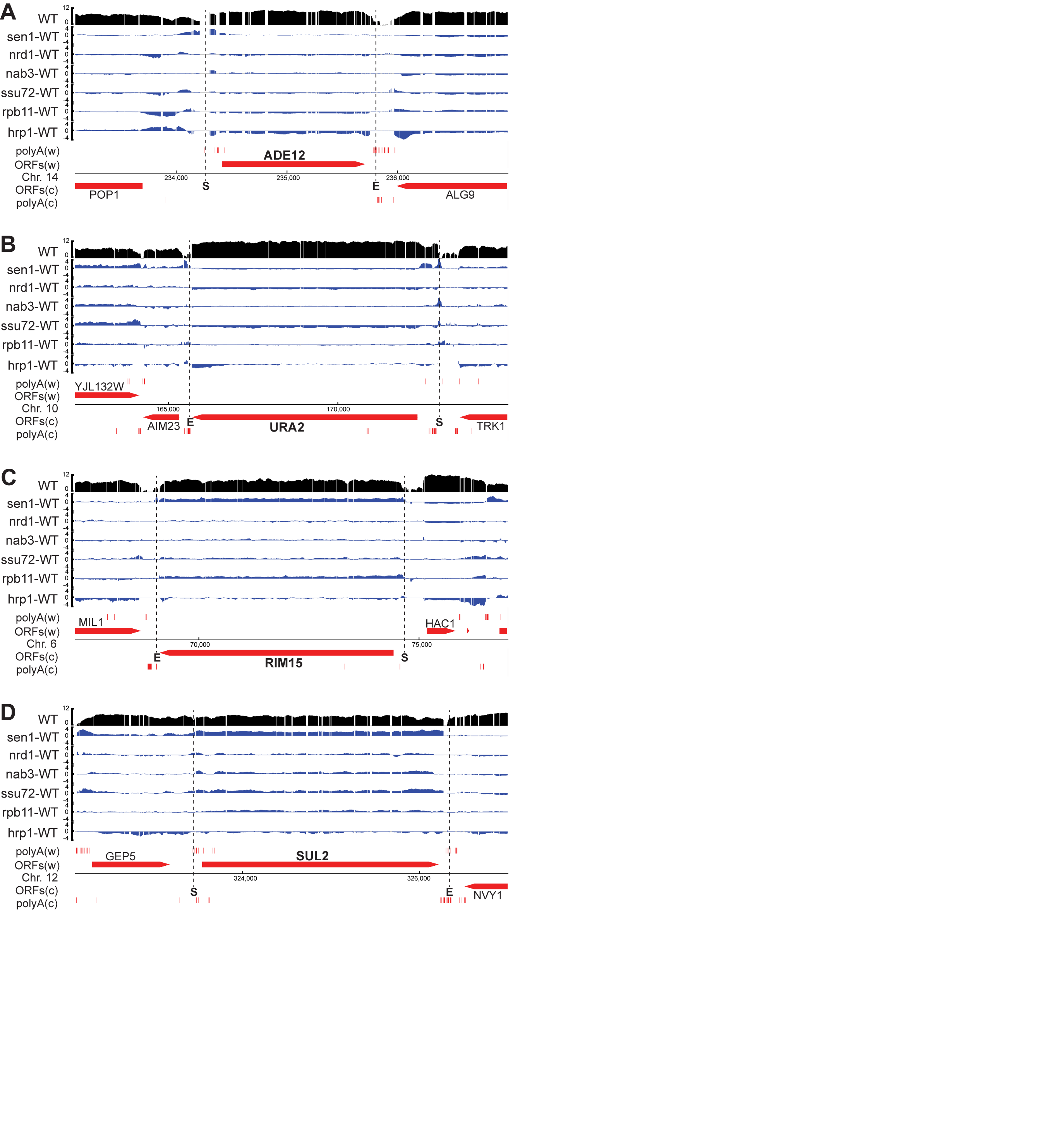

Supplement: S6 Fig — The microarray data are displayed as in Fig 5. (TIF) [file pgen.1006863.s006.tif]

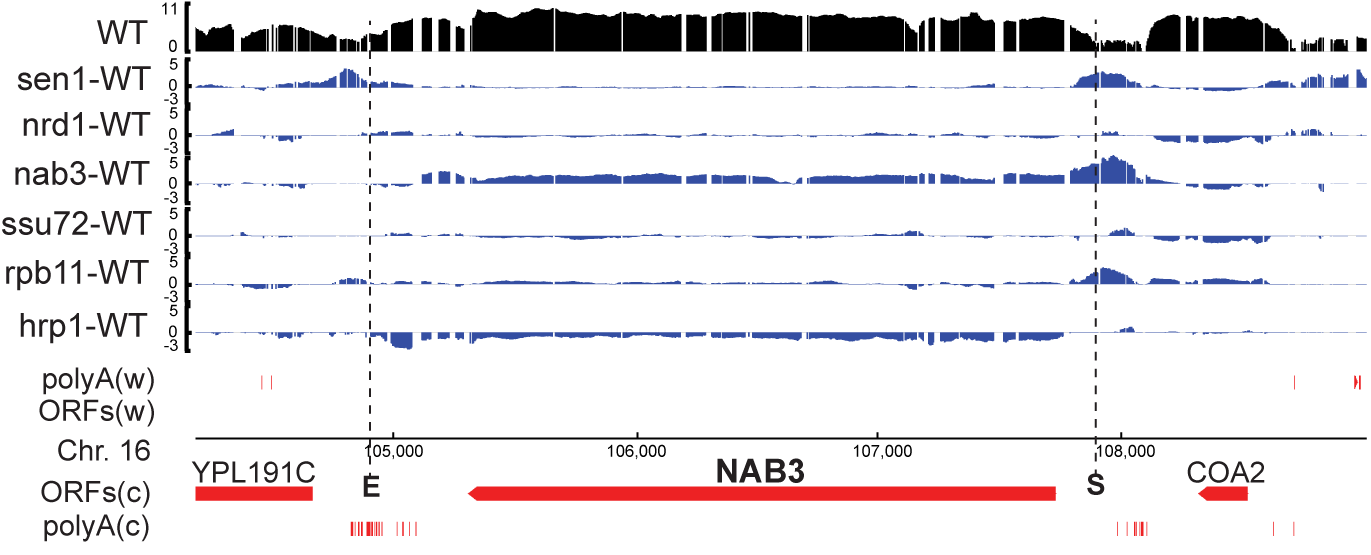

Supplement: S7 Fig — Changes in transcript levels over the indicated genes are displayed as in Fig 5. (TIF) [file pgen.1006863.s007.tif]

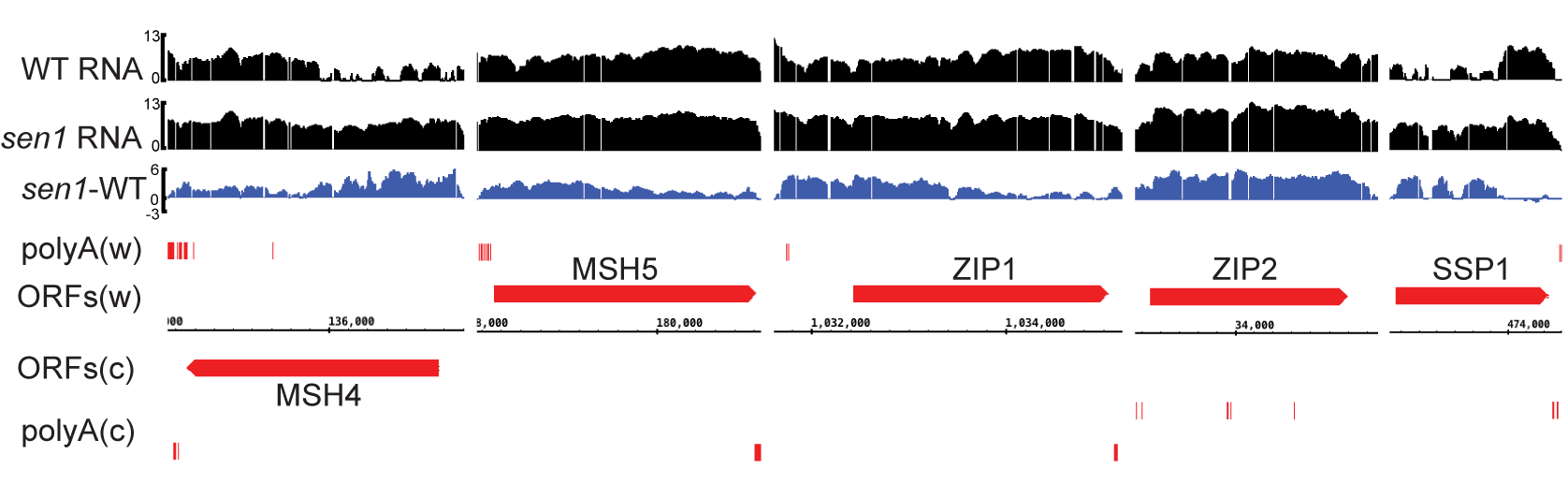

Supplement: S8 Fig — ZIP2 is the only gene shown that does not display an upstream bias in transcript accumulation in the sen1 mutant. (TIF) [file pgen.1006863.s008.tif]
