# Supplementary material for: Transcriptomes of six mutants in the Sen1 pathway reveal combinatorial control of transcription termination across the Saccharomyces cerevisiae genome
Source: PLoS Genet. 2017 Jun 30;13(6):e1006863. doi: 10.1371/journal.pgen.1006863 (PMC5513554; doi:10.1371/journal.pgen.1006863)
Supplement: S1 Table — Percent change in the transcript level downstream of the indicated sn/snoRNA genes in mutant strains relative to wild-type was calculated as described in the text. U RNAs transcribed from SNR genes are listed after a slash. Positive changes of greater than 10% are considered indicative of terminator read-through and are in bold font. Changes of greater than 100% are likely due to overlapping transcripts from other sources. (DOCX) [file pgen.1006863.s009.docx]

# Table S1. Differential read-through ratio (%) of sn/snoRNA genes in the six mutants.

| *SNR* gene | *sen1* | *nrd1* | *nab3* | *ssu72* | *rpb11* | *hrp1* |
| --- | --- | --- | --- | --- | --- | --- |
| *SNR3* | **66.68** | **16.01** | **59.17** | 9.63 | 5.76 | -3.95 |
| *SNR4* | **23.90** | **10.03** | **17.31** | **15.95** | 4.12 | **10.58** |
| *SNR5* | **34.22** | **14.96** | **13.92** | **19.12** | 8.45 | 3.80 |
| *SNR6/*U6 ^b^ | 5.86 | 0.82 | 0.86 | 1.11 | -0.04 | 1.32 |
| *SNR7/*U5 | 7.90 | 3.21 | **16.60** | 4.56 | 4.66 | 0.12 |
| *SNR8* | **35.80** | 0.05 | 0.75 | 3.99 | 2.71 | -0.06 |
| *SNR10* | **28.48** | **10.96** | -3.41 | 4.74 | 3.24 | -7.63 |
| *SNR11* | **72.94** | **34.20** | 4.61 | **32.55** | 8.10 | 9.64 |
| *SNR13* | **66.60** | **44.05** | **32.55** | **33.98** | 5.87 | 1.45 |
| *SNR14/*U4 | **36.37** | **48.92** | **21.03** | **25.58** | -10.66 | -14.27 |
| *SNR17a*/U3 | 3.41 | 1.43 | 1.01 | 2.02 | 0.70 | 0.27 |
| *SNR17b*/U3 | 7.14 | 9.92 | -1.49 | 4.92 | -1.82 | -0.25 |
| *SNR19/*U1 | 3.50 | 1.72 | 1.15 | 3.02 | 0.45 | 0.10 |
| *SNR20/*U2 | -0.24 | -0.42 | -1.24 | -0.21 | -2.80 | -3.62 |
| *SNR30* | **23.65** | 5.93 | 2.96 | 0.84 | 1.26 | 0.49 |
| *SNR31* | **175.48** | **24.73** | **18.91** | **29.31** | -2.15 | **23.18** |
| *SNR32* | **39.01** | 5.49 | 5.85 | 2.98 | 5.86 | 1.69 |
| *SNR33* | **342.00** | **91.84** | **55.18** | **253.01** | **143.24** | **55.28** |
| *SNR34* | **82.83** | **33.41** | **43.59** | 7.75 | 4.89 | 2.64 |
| *SNR35* | **57.01** | **15.59** | 2.20 | **11.27** | -1.08 | 7.51 |
| *SNR37* | **109.78** | **13.62** | **20.84** | 9.08 | 0.80 | 1.82 |
| *SNR39B* | **60.33** | 3.97 | 0.81 | 9.75 | 1.46 | -0.49 |
| *SNR40* | 1.92 | 0.00 | -0.23 | 0.27 | 3.52 | 0.07 |
| *SNR42* | **69.46** | **38.83** | **35.94** | **58.68** | -14.24 | **62.41** |
| *SNR45* | **68.59** | 6.12 | **17.20** | 2.31 | 4.43 | -3.91 |
| *SNR46* | **15.57** | 4.14 | 3.59 | 2.50 | 2.06 | -1.26 |
| *SNR47* | **131.89** | **28.24** | **86.95** | **58.17** | **36.07** | **11.44** |
| *SNR48* | **236.27** | **94.56** | **178.67** | **89.58** | **39.32** | **18.01** |
| *SNR49* | **46.84** | **42.25** | **10.06** | -116.76 | -85.00 | **209.31** |
| *SNR50* | **106.12** | 9.72 | 6.41 | **38.01** | 6.17 | 7.21 |
| *SNR51* ^a^ | **137.87** | **16.99** | -2.67 | **141.70** | **48.38** | **34.35** |
| *SNR61* ^a^ | **106.65** | -5.08 | **14.87** | **42.45** | 4.78 | **40.81** |
| *SNR63* | 8.02 | **10.88** | -3.81 | 6.07 | -0.11 | -3.21 |
| *SNR64* | **74.69** | **32.90** | **21.55** | **34.52** | 7.97 | 2.18 |
| *SNR69* | **45.31** | **26.09** | **31.69** | **22.07** | 6.40 | **48.53** |
| *SNR71* | **38.21** | **25.25** | **45.31** | **37.93** | **19.15** | **31.88** |
| *SNR72* | **85.43** | -10.29 | **12.56** | **39.28** | **60.65** | -9.02 |
| *SNR79* | **162.34** | 7.37 | **62.63** | **39.43** | **12.71** | 9.31 |
| *SNR80* | **161.12** | **60.90** | **81.84** | **115.87** | **36.91** | **140.46** |
| *SNR81* | **67.01** | 6.63 | **13.98** | **38.26** | **10.53** | -0.02 |
| *SNR82* | **124.83** | **41.30** | **14.26** | **45.11** | **57.60** | **87.55** |
| *SNR84* | **15.29** | **10.56** | **20.96** | **11.10** | -4.61 | **19.95** |
| *SNR85* | **98.99** | -2.51 | 1.45 | **10.06** | -0.24 | -0.84 |
| *SNR86* | **12.17** | 2.31 | -1.18 | 4.47 | -1.97 | -2.03 |
| *SNR87* | **86.90** | -16.44 | **37.79** | **71.02** | **21.89** | -49.28 |
| *SNR161* | **231.51** | **223.36** | **212.37** | **148.84** | **35.16** | **112.28** |
| *SNR189* | **62.67** | 0.33 | **7.24** | **27.15** | **9.35** | -0.38 |

^a^  snoRNA genes that are transcribed last in a polycistronic cluster.

^b^ Transcribed by RNA polymerase III.
