# Supplementary material for: Transcriptomes of six mutants in the Sen1 pathway reveal combinatorial control of transcription termination across the Saccharomyces cerevisiae genome
Source: PLoS Genet. 2017 Jun 30;13(6):e1006863. doi: 10.1371/journal.pgen.1006863 (PMC5513554; doi:10.1371/journal.pgen.1006863)
Supplement: S3 Table — Genes indicated in bold font are shown in Fig 7 or S8 Fig. (DOCX) [file pgen.1006863.s011.docx]

# Table S3. Genes induced in meiosis that are more than two-fold up-regulated in vegetative cells in the *sen1* mutant.

| Systematic Name | Standard Name | Fold increase in *sen1* |  | Systematic Name | Standard Name | Fold increase in *sen1* |
| --- | --- | --- | --- | --- | --- | --- |
| *YHR157W* | *REC104* | 22.98 |  | *YJL037W* | *IRC18* | 2.92 |
| *YGL183C* | *MND1* | 17.33 |  | *YBR186W* | *PCH2* | 2.87 |
| *YLR054C* | *OSW2* | 11.71 |  | *YDR113C* | *PDS1* | 2.81 |
| *YMR017W* | *SPO20* | 8.64 |  | *YLR341W* | *SPO77* | 2.81 |
| *YPL200W* | *CSM4* | 8.31 |  | *YHL022C* | *SPO11* | 2.81 |
| *YGL249W* | *ZIP2* | 8.24 |  | *YHR079C-A* | *SAE3* | 2.74 |
| *YBR148W* | *YSW1* | 7.18 |  | *YML128C* | *MSC1* | 2.72 |
| *YDR402C* | *DIT2* | 7.02 |  | *YFL003C* | *MSH4* | 2.66 |
| *YNL196C* | *SLZ1* | 6.95 |  | ***YOL104C*** | ***NDJ1*** | **2.63** |
| ***YBR250W*** | ***SPO23*** | **6.73** |  | *YAL068C* | *PAU8* | 2.60 |
| ***YOR351C*** | ***MEK1*** | **5.59** |  | *YGR225W* | *AMA1* | 2.57 |
| *YPL121C* | *MEI5* | 5.59 |  | *YNL128W* | *TEP1* | 2.57 |
| *YGR059W* | *SPR3* | 5.46 |  | *YJL106W* | *IME2* | 2.54 |
| *YLR329W* | *REC102* | 5.32 |  | ***YGL251C*** | ***HFM1*** | **2.49** |
| *YCL048W* | *SPS22* | 5.21 |  | *YLR343W* | *GAS2* | 2.42 |
| *YGL045W* | *RIM8* | 5.16 |  | *YER179W* | *DMC1* | 2.42 |
| *YOR298W* | *MUM3* | 5.11 |  | *YDR285W* | *ZIP1* | 2.37 |
| *YDR273W* | *DON1* | 4.66 |  | *YDR218C* | *SPR28* | 2.33 |
| *YOR190W* | *SPR1* | 4.60 |  | ***YHR184W*** | ***SSP1*** | **2.31** |
| *YOR313C* | *SPS4* | 4.58 |  | *YDL154W* | *MSH5* | 2.29 |
| *YOL047C* | *LDS2* | 4.45 |  | *YDR506C* | *GMC1* | 2.29 |
| *YGL033W* | *HOP2* | 4.31 |  | *YGL158W* | *RCK1* | 2.25 |
| *YER044C-A* | *MEI4* | 4.18 |  | *YLR308W* | *CDA2* | 2.21 |
| *YHR139C* | *SPS100* | 4.03 |  | *YLR445W* | *GMC2* | 2.17 |
| *YOR178C* | *GAC1* | 3.99 |  | *YLR213C* | *CRR1* | 2.15 |
| *YPL027W* | *SMA1* | 3.98 |  | *YHR014W* | *SPO13* | 2.15 |
| *YMR306W* | *FKS3* | 3.75 |  | *YDR523C* | *SPS1* | 2.15 |
| *YPL130W* | *SPO19* | 3.75 |  | *YMR133W* | *REC114* | 2.11 |
| *YPR007C* | *REC8* | 3.69 |  | *YBR045C* | *GIP1* | 2.11 |
| *YDR403W* | *DIT1* | 3.67 |  | *YJL038C* | *LOH1* | 2.11 |
| *YOL132W* | *GAS4* | 3.51 |  | *YOL091W* | *SPO21* | 2.05 |
| *YDR522C* | *SPS2* | 3.38 |  | *YPL164C* | *MLH3* | 2.05 |
| *YNL210W* | *MER1* | 3.33 |  | *YNL012W* | *SPO1* | 2.04 |
| *YDR260C* | *SWM1* | 3.02 |  | *YIL073C* | *SPO22* | 2.01 |
| *YER046W* | *SPO73* | 2.95 |  |  |  |  |
